# Assessing the impact of transcatheter edge-to-edge repair on reverse remodeling in secondary mitral regurgitation: a systematic review and meta-analysis

**DOI:** 10.3389/fcvm.2025.1714337

**Published:** 2026-01-30

**Authors:** Adolf Lichtfusz, Nina Galdzytska, Dorottya Gergő, Bence Szabó, Péter Hegyi, Zsolt Molnár, Gábor Duray, Judit Papp

**Affiliations:** 1 Centre for Translational Medicine, Semmelweis University, Budapest, Hungary; 2 Department of Cardiology, Central Hospital of Northern Pest – Military Hospital, Budapest, Hungary; 3 Department of Cardiac Surgery, Central Hospital of Northern Pest – Military Hospital, Budapest, Hungary; 4 Department of Pharmacognosy, Semmelweis University, Budapest, Hungary; 5 Institute for Translational Medicine, Medical School, University of Pécs, Pécs, Hungary; 6 Institute of Pancreatic Diseases, Semmelweis University, Budapest, Hungary; 7 Department of Anesthesiology and Intensive Therapy, Semmelweis University, Budapest, Hungary; 8 Department of Anesthesiology and Intensive Therapy, Poznan University of Medical Sciences, Poznan, Poland; 9 Heart and Vascular Center, Semmelweis University, Budapest, Hungary

**Keywords:** echocardiagraphy, edge to edge repair, heart faiIure, reverse remodeling, secondary mitral regurgitation (SMR)

## Abstract

**Background and aim:**

Transcatheter edge-to-edge repair (TEER) is a minimally invasive approach to reduce secondary mitral regurgitation (SMR) in patients with heart failure. However, there is limited evidence on its effectiveness in achieving reverse remodeling. Our aim was to assess the effects of TEER over time and to compare the effects of TEER plus GDMT vs. GDMT alone on echocardiographic parameters.

**Methods:**

A systematic search of MEDLINE, EMBASE, and CENTRAL was conducted from inception to November 16, 2023. Eligible studies included patients with SMR treated with TEER and echocardiographic follow-ups. We evaluated changes in left ventricular end-diastolic diameter (LVEDD), left ventricular end-systolic diameter (LVESD), left ventricular end-diastolic volume (LVEDV), left ventricular end-systolic volume (LVESV), left atrial volume (LAV), left ventricular ejection fraction (LVEF) and NTproBNP levels.

**Results:**

Of 9,290 identified studies, 38 met inclusion criteria. After TEER, statistically significant reductions were observed in LVEDD (–1.63 mm), LVESD (–1.20 mm), LVEDV (–14.21 mL), and LVESV (–9.24 mL). Changes in LAV (–5.70 mL) and LVEF (+1.10%) were not statistically or clinically meaningful. NT-proBNP decreased substantially (–1,340 pg/mL). In comparative analyses, TEER plus GDMT did not show statistically significant differences vs. GDMT alone for any parameter, including LVEDD (–1.02 mm), LVEDV (–11.98 mL), LVEF (–0.14%), and LVESV (–5.29 mL). TEER reduced grade 3–4 MR from 99% to 9%.

**Systematic Review Registration:**

identifier CRD42023483404.

**Conclusion:**

TEER results in statistically significant but clinically small changes in echocardiographic parameters, and no clear advantage over GDMT alone. These findings should be interpreted with caution given the high heterogeneity and low certainty of evidence. Further studies are needed to define which SMR patient subgroups may derive meaningful reverse-remodeling benefit from TEER.

**Systematic Review Registration:**

https://www.crd.york.ac.uk/prospero/display_record.php?RecordID=483404, PROSPERO CRD42023483404.

## 1 Introduction

Heart Failure (HF) affects more than 64 million people worldwide (1). It encompasses changes in cardiac structure, such as myocardial composition, myocyte deformation, and numerous biochemical and molecular alterations affecting heart function and reserve capacity (2). These collective changes are known as cardiac remodeling. A major objective in the treatment of HF is to stop or reverse this process, the latter called reverse remodeling (3). Reverse remodeling is characterized by a reduction in chamber volumes and mass, recovery of ventricular shape, as well as improvement in ejection fraction and is often accompanied by enhanced *β*-adrenergic and heart-rate responsiveness (4, 5).

Mitral regurgitation (MR) is the most common valvular heart disease in HF patients. MR affects almost 65% of patients with chronic HF and 50% of those with acute HF (6). MR has primary and secondary forms. Primary MR occurs due to structural abnormalities of the mitral valve apparatus itself. In contrast, secondary mitral regurgitation (SMR) occurs due to alterations in the anatomy of left ventricle (ischemic etiology or cardiomyopathy) or left atrium (most commonly due to atrial fibrillation), causing displacement of the papillary muscles and alteration of the geometry of the mitral annulus, leading to leaflet tethering and malcoaptation (7). Severe SMR in heart failure leads to disease progression, increasing mortality, and hospitalization rates (8). This is due to volume overload in the left atrium and ventricle, elevated pulmonary pressures, reduced forward cardiac output, and subsequent deterioration of left ventricular function. These hemodynamic disturbances contribute to pulmonary congestion, decreased perfusion of vital organs, arrhythmias, and further structural remodeling of the heart, perpetuating a cycle of deteriorating cardiac performance and clinical decline (9). Managing severe SMR in HF patients requires a tailored approach by the heart team that balances the benefits and risks of surgical vs. transcatheter interventions (10). Traditional surgical methods, such as mitral valve repair, are recommended for patients undergoing coronary artery bypass grafting (CABG) or other cardiac surgeries (11).

Transcatheter edge-to-edge repair (TEER) with the MitraClip and Pascal systems has emerged as a minimally invasive alternative for patients with severe SMR (12).

Although several studies have shown that TEER can alleviate symptoms and increase functional capacity, thereby improving the quality of life of these patients, data on its effectiveness in achieving reverse remodeling are lacking (13). Therefore, this study aims to investigate the effectiveness of reverse remodeling of the TEER technique compared to guideline-directed medical therapy (GDMT). Understanding the impact of TEER on cardiac reverse remodeling may lead to optimized treatment strategies and improved outcomes for patients suffering from secondary mitral regurgitation and heart failure.

## 2 Methods

### 2.1 Study design

In this systematic review and meta-analysis, we evaluated the effects of reverse remodeling of transcatheter edge-to-edge repair (TEER) of the mitral valve. The study was divided into two parts. First, we quantified reverse remodeling by assessing changes in echocardiographic parameters, including left ventricular end-diastolic diameter (LVEDD), left ventricular end-systolic diameter (LVESD), left ventricular end-diastolic volume (LVEDV), left ventricular end-systolic volume (LVESV), left atrial volume (LAV), and ejection fraction (EF), measured from baseline to follow-up. Second, we compared these changes between patients receiving guideline-directed medical therapy alone and those receiving GDMT plus TEER.

### 2.2 Protocol and search strategy

The study protocol was registered with PROSPERO (CRD42023483404) with no deviations. We adhered to the PRISMA guidelines in reporting our results. We searched MEDLINE (via PubMed), EMBASE, and Cochrane CENTRAL for eligible articles without restrictions up to November 16, 2023. The search strategy focused on mitral transcatheter edge-to edge repair and mitral regurgitation.

### 2.3 Eligibility criteria

We included randomized controlled trials (RCTs) and cohort studies, excluding conference abstracts, editorials, case reports, case series, non-peer-reviewed articles, and animal experiments. Studies were selected to align with our predefined PICO frameworks, assessing echocardiographic parameters before and after follow-up in one part and comparing TEER + GDMT with GDMT alone in the other. Subgroup analyses were conducted by follow-up period, including post-discharge, one-month, three-month, six-month, twelve-month, and mixed follow-up periods.

### 2.4 Study selection and data extraction

The search results were imported into EndNote 21 (Clarivate Analytics) for duplicate removal. Two independent authors (AL, NG) screened the remaining articles by title, abstract, and full text using Rayyan (14). In addition, we performed backward citation chasing. Cohen's kappa coefficient (*κ*) was calculated to assess inter-reviewer reliability, and disagreements were resolved by a third reviewer (DG). Data were extracted independently by two investigators (AL, NG) into a Microsoft Excel table, focusing on LVEDD, LVESD, LVEDV, LVESV, LAV, EF, NT-proBNP levels, and mitral regurgitation grade. In addition, we collected data on study details, patient demographics, and relevant echocardiographic outcomes.

### 2.5 Statistical analysis

A random-effects model was used to pool effect sizes, with three studies as a minimum. The following effect size measures were pooled: mean difference (MD) of changes from baseline for LVEDD, LVESD, LVEDV, LVESV, LAV, EF, and NT-proBNP levels when comparing medical therapy with combined therapy; single means when assessing the change in echocardiographic parameters before and after surgical therapy; and proportions for the changes in mitral regurgitation grades. Pooled effect sizes were expressed as point estimates and 95% confidence interval. The inverse variance weighting method was used to calculate the pooled MD. To estimate the heterogeneity variance measure (*τ*2), the restricted maximum-likelihood estimator was used with the Q profile method for confidence intervals (15, 16). The t-distribution-based method was used to calculate CI of MD of individual studies. A random intercept logistic regression model was used to pool outcomes [as recommended by Schwarzer et al. (17) and Stijnen et al. (18)]. The maximum likelihood method was used to estimate the heterogeneity variance measure (*τ*^2^). In forest plots, the Clopper-Pearson method (19) was used to calculate the CI of proportion of individual studies. We used a Hartung-Knapp adjustment for Cis (20, 21). In addition, between-study heterogeneity was described by the Higgins & Thompson's *I*^2^ statistics (22).

For subgroup analysis, we used a fixed-effects “plural” model (aka. mixed-effects model). To assess the difference between the subgroups, we used a “Cochrane *Q*” test (an omnibus test) (16). All statistical analyses were performed with R (R Core Team 2021, v4.1.2) using the meta (Schwarzer 2022, v6.1.0 28) (23) package for basic meta-analysis calculations and plots, and dmetar (Cuijpers, Furukawa, and Ebert 2023, v0.0.9000) (24) package for additional influential analysis calculations and plots. For additional details on calculations, data synthesis and publication bias assessment, see the Supplementary Material.

### 2.6 Risk of bias assessment

On the basis of the recommendation of the Cochrane Collaboration, two investigators (AL, NG) independently assessed the risk of bias for each outcome using the ROBINS-I tool for cohort studies with seven domains, and the ROB2 tool for randomized control studies with five domains (25, 26). Disagreements were resolved by consensus. Risk of bias was assessed independently for each outcome, in accordance with ROBINS-I guidance and as prespecified in the PROSPERO protocol.

### 2.7 Quality of evidence

Certainty of evidence was assessed according to the Grading of Recommendations Assessment, Development, and Evaluation (GRADE) recommendation (27). Two independent investigators (AL, NG) evaluated all criteria for all outcomes, and disagreements were resolved by consensus.

## 3 Results

### 3.1 Literature search and study selection

**Figure 1:**
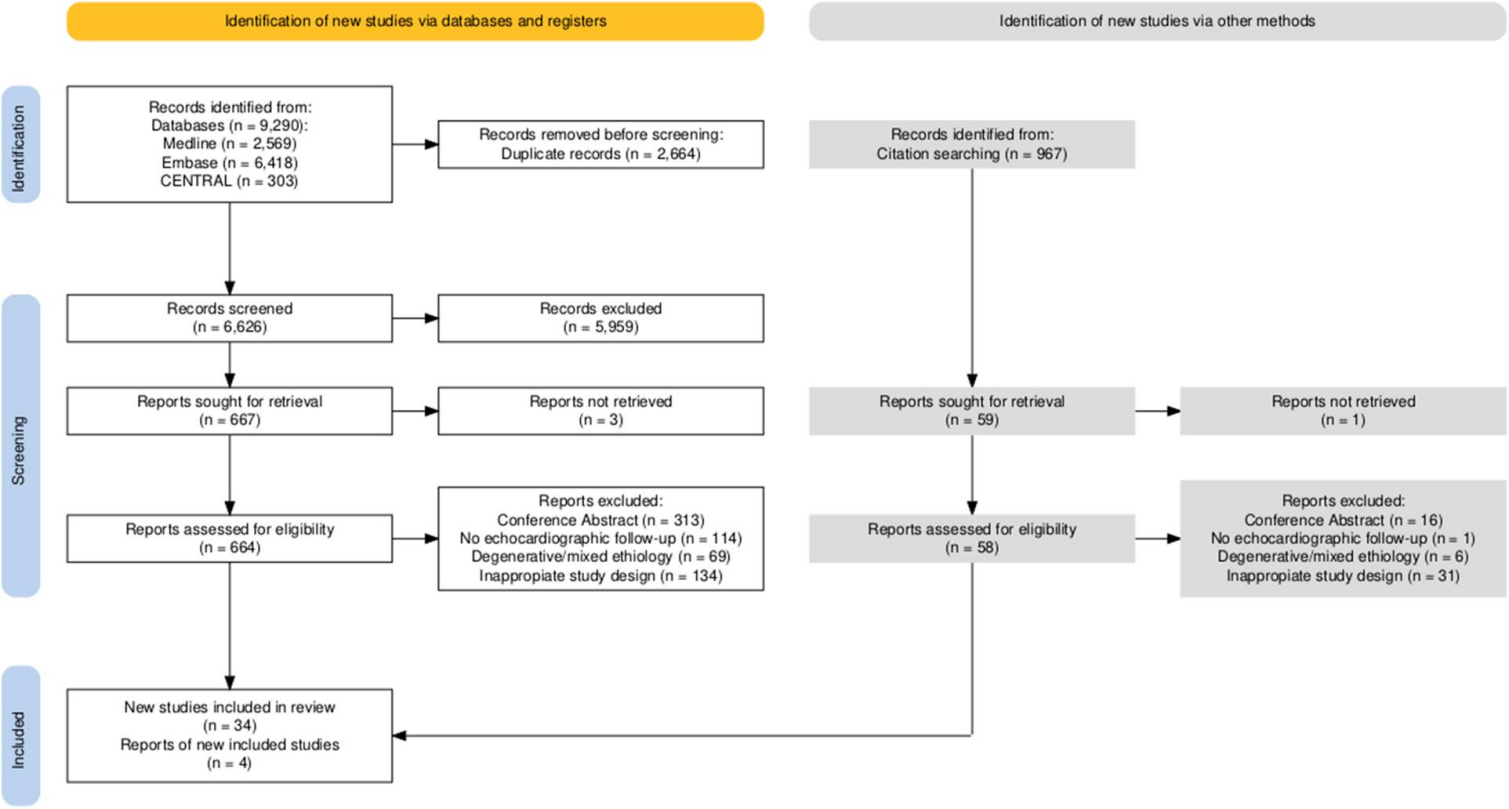
Details of the search and selection are illustrated in the PRISMA flow chart.

Altogether, 9290 studies were identified with our search key in the three main online databases. The search key resulted in 2569 hits on MEDLINE (via Pubmed), 6418 on EMBASE, and 303 on CENTRAL (via Cochrane Library). After duplicate removal, 6626 records remained for title and abstract selection. A total of 667 studies were collected for full-text selection, of which 3 records were not found. After full-text selection, 34 eligible studies remained. After full-text selection, we performed backward citation chasing and found another four studies (Figure 1).

### 3.2 Study characteristics

There was one article that was an RCT (MitraFR trial) (12) and one was a *post hoc* analysis of the COAPT RCT trial (28), the rest of the studies were retrospective cohort studies. The main follow-up periods of the investigations were 1, 6, and 12 months. The studies included were conducted in different countries, with widely varied sample sizes. Between-study heterogeneity varied according to different follow-up durations, baseline patient characteristics, and study design. Additional clinically relevant baseline parameters, including MR severity, GDMT optimization, and CRT use, are presented in Supplementary Supplementary Tables S5 and S6.

Baseline characteristics of studies evaluating baseline-to-follow-up changes are presented in Table 1, while studies comparing TEER plus GDMT with GDMT alone are summarized in Table 2.

**Table 1:** Baseline characteristics of studies investigating TEER + GDMT effect overtime.

First author	Intervention age	Men intervention (%)	Diabetes mellitus intervention (%)	History of atrial fibrillation intervention (%)	Patient number at baseline	Patient number at follow-up
Ailawadi et al. (45)	73.3 ± 10.5	352 (59.1%)	232 (38.9%)	388 (65%)	597	402
Albini et al. (46)	79.7 ± 7.5	12 (92%)	5 (38%)	9 (69%)	13	13
Altiok et al. (47)	73 ± 9	24 (62%)	—	—	36	36
Barth (LVEF < 20) et al. (7)	68.9 ± 8.0	12 (100%)	6 (50%)	10 (83%)	12	11
Barth (LVEF > 20) et al. (7)	74.7 ± 7.9	44 (70%)	20 (32%)	44 (70%)	63	53
Barth (PASCAL) et al. (48)	78.8 ± 8	40 (63.5%)	13 (20.6%)	46 (73.0%)	38	38
Berardini et al. (49)	67 ± 11	58 (77%)	29 (39%)	—	68	68
Buck et al. (50)	71.7 ± 11.1	34 (75.5%)	—	—	45	45
Chan et al. (51)	71 ± 14	9 (75%)	—	—	12	10
Cimino et al. (52)	73 ± 7.7	22 (48%)	11 (24%)	10 (22%)	41	41
Citro et al. (53)	72.5 ± 9.6	26 (63.4%)	14 (34.1%)	20 (48.7%)	41	41
Demir et al. (54)	—	—	—	—	122	122
El Shurafa et al. (55)	67 (56.5–72.5)	54 (61.36%)	57 (64.77%)	17 (19.32%)	88	69
Giaimo et al. (56)	70.3 ± 8.4	23 (73.6%)	6 (20%)	14 (46.7%)	29	21
Giannini et al. (57)	75 (63–81)	23 (65.7%)	9 (27.5%)	18 (51.4%)	35	35
Godino et al. (58)	73 ± 8	50 (83%)	18 (30%)	21 (35%)	53	53
González et al. (59)	68.2 ± 10.9	82 (88.2%)	26 (28.0%)	49 (52.7%)	93	93
Hagnas (decreased LVEF) et al. (60)	72.7 ± 8.4	190 (60%)	133 (42%)	141 (44%)	399	399
Hagnas (improved LVEF) et al. (60)	72.9 ± 11.8	23 (56%)	11 (27%)	29 (71%)	399	399
Hagnas (unchanged LVEF) et al. (60)	71.9 ± 8.9	26 (65%)	10 (25%)	15 (38%)	399	399
Han Yoon (a-FMR) et al. (61)	78.8 ± 9.7	51 (44%)	35 (30.2%)	84 (72.4%)	116	116
Han Yoon (v-FMR) et al. (61)	72.1 ± 12.8	313 (62%)	169 (33.5%)	265 (52.5%)	505	505
Kamperidis et al. (62)	72 ± 10	11 (50%)	9 (43%)	10 (48%)	22	22
Nickenig et al. (63)	72.8 ± 9.8	179 (67.7%)	87 (33.1%)	—	264	264
Nita (LVRR) et al. (64)	78.2 ± 6.4	49 (60.5%)	18 (22.2%)	47 (58.0%)	53	53
Nita (no LVRR) et al. (64)	75.6 ± 10.2	62 (74.7%)	25 (30.1%)	58 (69.9%)	58	58
Ohno (Moderate/Severe TR) et al. (65)	73.2 ± 6.4	27 (57.4%)	22 (46.8%)	22 (46.8%)	26	26
Ohno (None/Mild TR) et al. (65)	70.8 ± 9.9	66 (66.7%)	35 (35.4%)	34 (34.3%)	73	73
Orban et al. (66)	74.7 ± 10.1	121 (58.4%)	61 (29.5%)	—	207	207
Öztürk et al. (67)	77.6 ± 9.1	29 (58%)	14 (28%)	—	50	43
Palmiero et al. (68)	67.8 ± 9.0	—	—	—	25	25
Perl et al. (69)	69.3 ± 15.9	9 (90%)	4 (40%)	—	10	9
Scandura et al. (70)	73.0 ± 6.1	25 (83.3%)	14 (46.6%)	12 (40%)	30	30
Shechter et al. (71)	69 (55–76)	64 (66.7%)	32 (33.3%)	42 (43.8%)	96	43
Taramasso et al. (72)	68.4 ± 9.2	43 (82%)	14 (26.9%)	37 (17.3%)	52	50
Tay et al. (73)	70.4 (10.1)	60 (68.2%)	33 (37.5%)	42 (47.7%)	78	78
Toprak et al. (74)	58.2 ± 11.96	21 (75%)	5 (18%)	4 (14%)	19	19
Vitarelli et al. (75)	79.4 ± 5.5	18 (56.25%)	13 (40.6%)	6 (18.7%)	32	32

**Table 2:** Baseline characteristics of studies comparing TEER + GDMT to GDMT alone.

First author (Publication year)	Intervention age	Control age	Men intervention (%)	Men control (%)	History of atrial fibrillation intervention (%)	History of atrial fibrillation control (%)	Number of patients at baseline intervention	Number of patients at follow-up intervention	Number of patients at baseline control	Number of patients at follow-up control
Asch et al. (28)	—	—	—	—	—	—	281	281	295	295
Freixa et al. (76)	72.1+−7	67.2 ± 6	13 (81%)	12 (80%)	9 (56%)	5 (33%)	15	15	15	15
Hubert et al. (77)	70.0 ± 10.6	74.3 ± 9.7	—	—	14 (37.8%)	27 (47.4%)	37	32	19	16
Krawczyk-Ożóg et al. (78)	71.8 ± 7.8	73.0 ± 11.5	—	—	16 (60%)	16 (60.9%)	8	8	19	19
Obadia et al. (12)	70.1 ± 10.1	70.6 ± 9.9	120 (78.9%)	107 (70.4%)	52 (34.5%)	48 (32.7%)	152	86	152	76
Papadopoulos et al. (79)	72 ± 10	71 ± 11	42 (72.4%)	25 (86.2%)	28 (49.1%)	32 (37.5%)	58	58	28	28

### 3.3 Baseline to follow-up changes in echocardiographic parameters (TEER plus GDMT)

In the first part of our analysis, we assessed changes in echocardiographic parameters from baseline to follow-up in patients who underwent TEER procedure in addition to GDMT. We analyzed important echocardiographic parameters such as LVEDD, LVESD, LVEDV, LVESV, LAV and EF.

**Figure 2:**
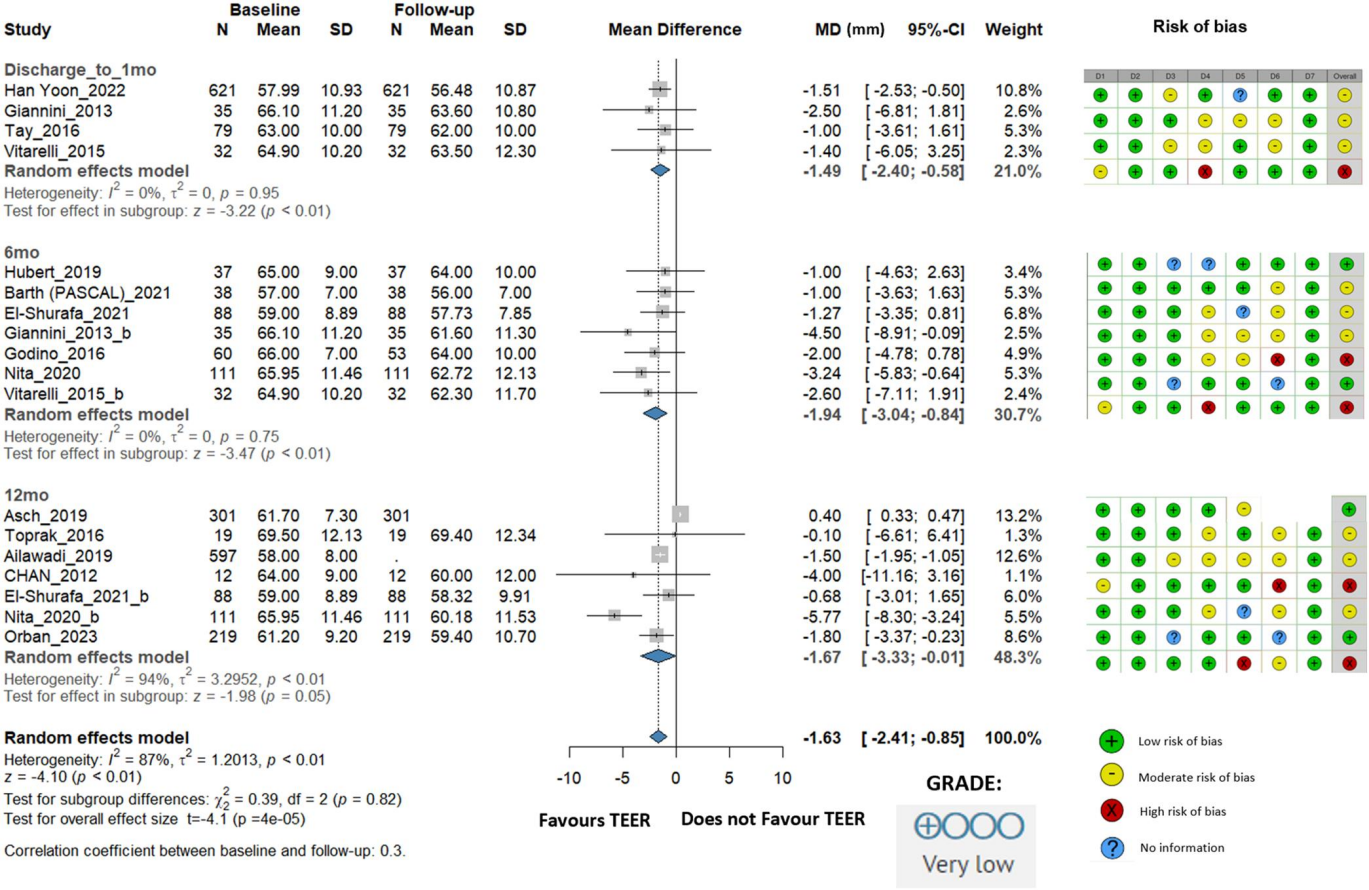
Effect of TEER + GDMT on left ventricular end diastolic diameter—1 month, 6 month, 12 month follow-up. MD, mean difference; CI, confidence interval; SD, standard deviation; D1, bias due to confounding; D2, bias in selection of participants; D3, bias in classification of interventions; D4, bias due to deviations from intended interventions; D5, bias due to missing data; D6, bias in measurement of outcomes; D7, bias in selection of reported result.

LVEDD showed a statistically significant reduction of −1.63 mm (95% CI: −2.41 to −0.85, *I*^2^ = 87%). At discharge/1 month, the change was −1.49 mm (95% CI: −2.40 to −0.58, *I*^2^ = 0%); at 6 months, where the most considerable change was observed, it was −1.94 mm (95% CI: −3.04 to −0.84, *I*^2^ = 0%), and at 12 months, the change was −1.67 mm (95% CI: −3.33 to −0.01, *I*^2^ = 94%) (Figure 2).

We also found a significant overall change in the mean LVESD of −1.20 mm (95% CI: −1.97 to −0.43, *I*^2^ = 33%). At discharge/1 month, the change was −0.56 mm (95% CI: −1.65 to 0.53, *I*^2^ = 0%). At 6 months, the change was more pronounced at −2.39 mm (95% CI: −3.77 to −1.00, *I*^2^ = 0%), whereas at 12 months, the change was −0.86 mm (95% CI: −2.26 to 0.54, *I*^2^ = 70%). The mixed-length follow-up showed a change of −1.89 mm (95% CI: −3.90 to 0.13, *I*^2^ = 0%) (Supplementary Figure S1).

**Figure 3:**
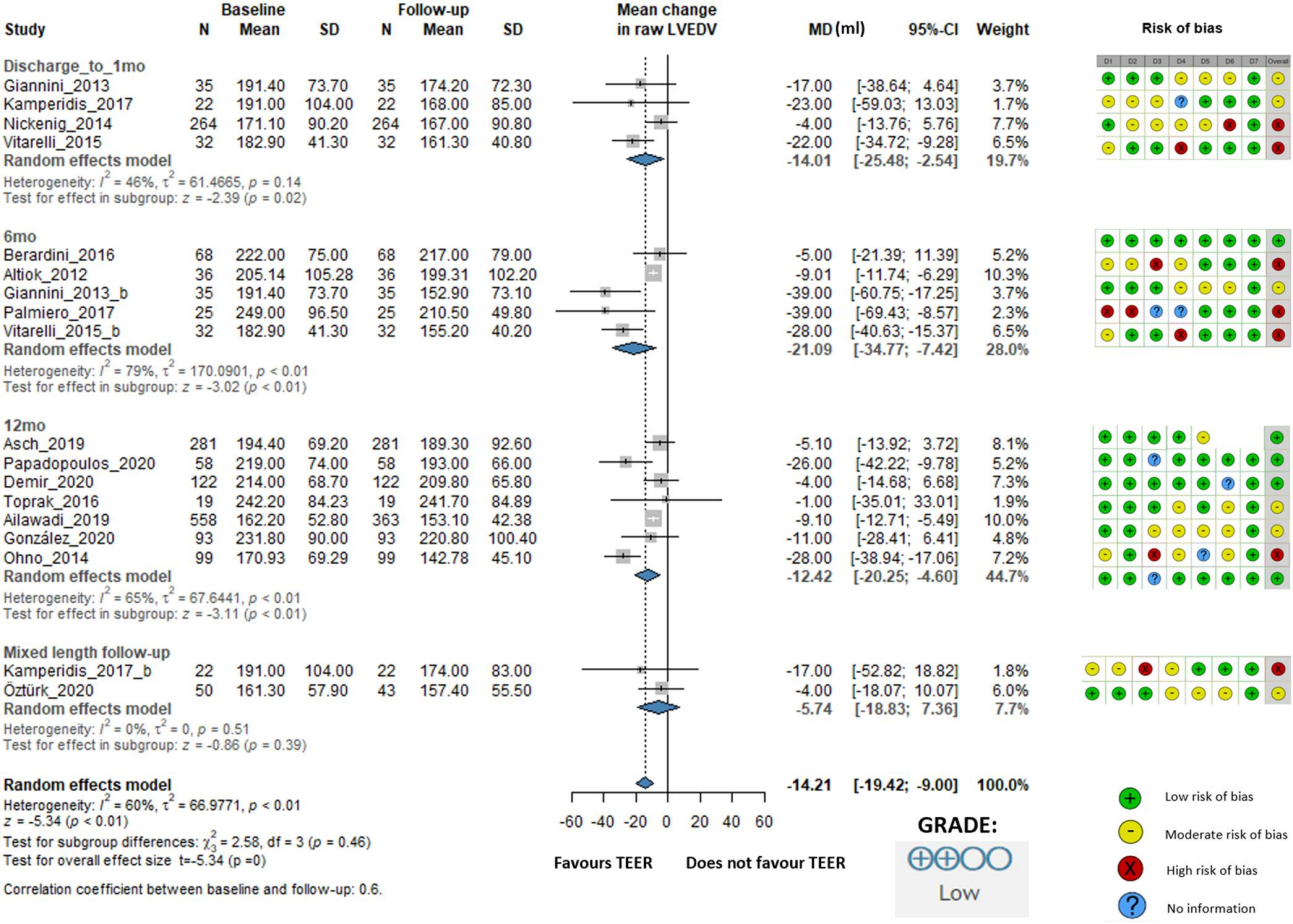
Effect of TEER + GDMT on left ventricular end-diastolic volume—1 months, 6 months, 12 months, and mixed-length follow-up. MD, mean difference; CI, confidence interval; SD, standard deviation; D1, bias due to confounding; D2, bias in selection of participants; D3, bias in classification of interventions; D4, bias due to deviations from intended interventions; D5, bias due to missing data; D6, bias in measurement of outcomes; D7, bias in selection of reported result.

LVEDV changed statistically significant, the overall change was −14.21 mL (95% CI: −19.42 to −9.00, *I*^2^ = 60%). At discharge/1 month, the change was −14.01 mL (95% CI: −25.48 to −2.54, *I*^2^ = 46%), at 6 months, it was −21.09 mL (95% CI: −34.77 to −7.42, *I*^2^ = 79%), and at 12 months, it was −12.42 mL (95% CI: −20.25 to −4.60, *I*^2^ = 65%). The mixed-length follow-up subgroup showed a change of −5.74 mL (95% CI: −18.83 to 7.36, *I*^2^ = 0%), indicating no statistically significant difference (Figure 3).

Studies providing LVEDVi showed an overall change of −4.08 mL/m^2^ (95% CI: −10.26 to 2.10, *I*^2^ = 74%), indicating no statistically significant difference. At discharge/1 month, the change was −2.12 mL/m^2^ (95% CI: −17.68 to 13.43, *I*^2^ = 62%). At 6 months, the change was −7.96 mL/m^2^ (95% CI: −16.10 to 0.17, *I*^2^ = 73%). However, at 12 months, there was an increase of 8.95 mL/m^2^ (95% CI: 2.26 to 15.64, *I*^2^ = 0%). Only two studies were available in the mixed-length follow-up subgroup, which showed a change of −11.60 mL/m^2^ (95% CI: −23.41 to 0.20, *I*^2^ = 0%) (Supplementary Figure S2).

LVESV showed an overall statistically significant change of −9.24 mL (95% CI: −14.00 to −4.48, *I*^2^ = 69%). At discharge/1 month, the change was −8.31 mL (95% CI: −16.61 to −0.02, *I*^2^ = 22%). At 6 months, the change was −13.25 mL (95% CI: −23.82 to −2.69, *I*^2^ = 73%), whereas at 12 months, the change was −8.42 mL (95% CI: −16.11 to −0.73, *I*^2^ = 78%). The mixed-length follow-up subgroup showed a change of −0.89 mL (95% CI: −13.92 to 12.15, *I*^2^ = 0%) (Supplementary Figure S3).

For LAV, the overall change was −5.70 mL (95% CI: −15.75 to 4.35, *I*^2^ =98%) demonstrating no statistically significant difference. At discharge/1 month, the change was −14.36 mL (95% CI: −25.23 to −3.50, *I*^2^ = 77%); at 6 months, it was −13.23 mL (95% CI: −20.00 to −6.47, *I*^2^ = 68%). At 12 months, it was −2.11 mL (95% CI: −14.56 to 10.34, *I*^2^ = 96%). These changes suggest a more beneficial effect on reversing left atrial remodeling during short-term follow-up (Supplementary Figure S4).

We also looked at LAVi, which showed an overall decrease of −6.86 mL/m^2^ (95% CI: −12.79 to −0.94, *I*^2^ = 98%), which, despite the statistical significance, did not reach clinical relevance (Supplementary Figure S5).

**Figure 4:**
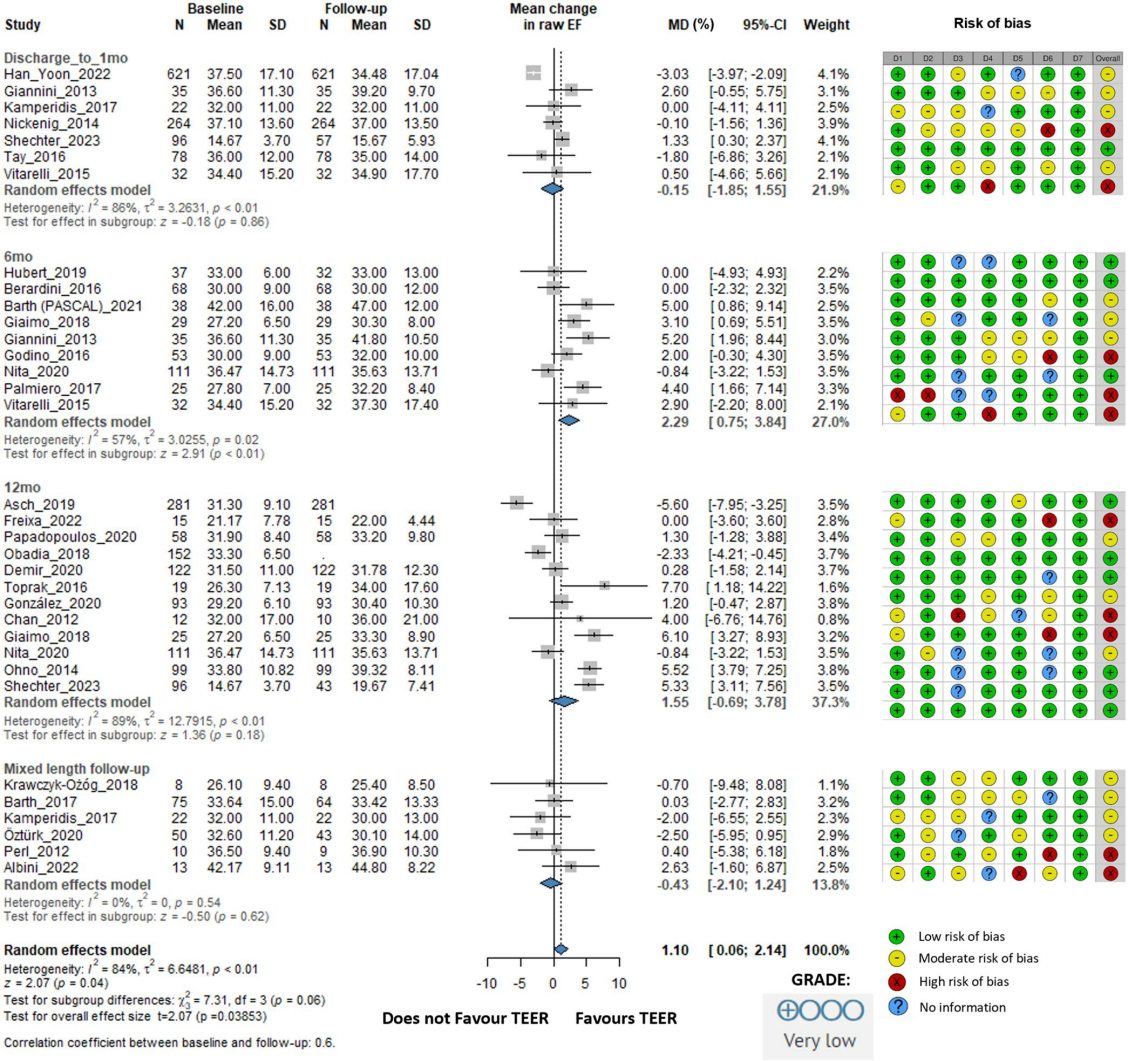
Effect of TEER + GDMT on ejection fraction—1 month, 6 months, 12 months, and mixed-length follow-up. MD, mean difference; CI, confidence interval; SD, standard deviation; D1, bias due to confounding; D2, bias in selection of participants; D3, bias in classification of interventions; D4, bias due to deviations from intended interventions; D5, bias due to missing data; D6, bias in measurement of outcomes; D7, bias in selection of reported result.

There was no improvement in ejection fraction either statistically or clinically. The overall change was 1.10% (95% CI: 0.06–2.14, *I*^2^ = 84%). At 6 months, EF improved by 2.29% (95% CI: 0.75–3.84, *I*^2^ = 57%). At 12 months, the EF improvement was 1.55% (95% CI: −0.69 to 3.78, *I*^2^ = 89%). In the mixed-length follow-up subgroup, EF slightly decreased by −0.43% (95% CI: −2.10 to 1.24, *I*^2^ = 0%) (Figure 4).

**Figure 5:**
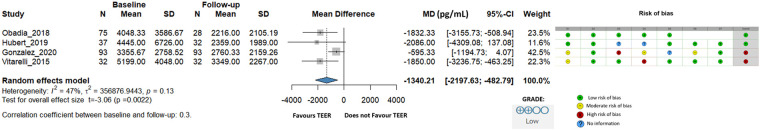
Effect of TEER + GDMT on NT-proBNP. MD, mean difference; CI, confidence interval; SD, standard deviation; D1, bias due to confounding; D2, bias in selection of participants; D3, bias in classification of interventions; D4, bias due to deviations from intended interventions; D5, bias due to missing data; D6, bias in measurement of outcomes; D7, bias in selection of reported result.

We also evaluated NT-proBNP levels, although data were only available from four studies with 237 individual patients, our analysis showed a remarkable decrease with an overall change of −1,340.21 pg/mL (95% CI: −2,197.63 to −482.79, *I*^2^ = 47%) (Figure 5).

The grade 3 mitral regurgitation proportion was 99% before TEER and 9% after the procedure showing the technical success of the procedure (Supplementary Figures S6a,b).

### 3.4 Comparison of GDMT to TEER plus GDMT

In the second part of our analysis, we compared changes in echocardiographic parameters between patients who received GDMT alone and those who underwent the TEER procedure in addition to GDMT.

**Figure 6:**
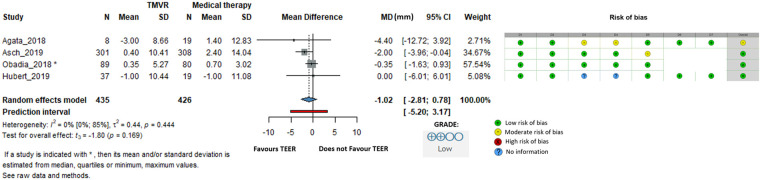
Effect of TEER + GDMT compared to GDMT alone on left ventricular end diastolic diameter. MD, mean difference; CI, confidence interval; SD, standard deviation; D1, bias due to confounding; D2, bias in selection of participants; D3, bias in classification of interventions; D4, bias due to deviations from intended interventions; D5, bias due to missing data; D6, bias in measurement of outcomes; D7, bias in selection of reported result.

When LVEDD was examined, the difference was −1.02 mm (95% CI: −2.81 to 0.78, *I*^2^ = 0%), indicating no statistically significant difference between groups (Figure 6).

The difference in ejection fraction was −0.14% (95% CI: −3.07 to 2.79, *I*^2^ = 63%), suggesting that additional TEER to GDMT did not improve ejection fraction (Supplementary Figure S7).

For the LVEDV, the difference was −11.98 mL (95% CI: −54.22 to 30.26, *I*^2^ = 80%), that was not statistically significant (Supplementary Figure S8). The LVESV difference was −5.29 mL (95% CI: −28.55 to 17.98, *I*^2^ = 44%), supporting TEER, but not in a statistically significant manner (Supplementary Figure S9).

### 3.5 Risk of bias and level of evidence certainty assessments

The risk of bias evaluation for the studies included is described in the accompanying figures (Figures 2–6; Supplementary Figures S1–S8), which show a low to moderate risk of bias across the majority of studies. Although several studies revealed possible issues, such as missing outcome data or unclear blinding procedures, the overall evaluation indicates that the risk of bias was not significant enough to undermine the reliability of the results. The results of the GRADE assessment of the level of evidence certainty are presented in Supplementary Tables S2 and S4.

### 3.6 Heterogeneity and publication bias

Across the main outcomes, between-study heterogeneity was substantial, with wide confidence intervals indicating considerable uncertainty. The largest contributors to heterogeneity were differences in sample size, variability in effect sizes, and the markedly different confidence-interval widths across studies. Formal assessment of publication bias was limited because only a few outcomes were informed by more than ten studies, which restricts the reliability and interpretability of funnel-plot-based methods according to Cochrane recommendations. Therefore, although no obvious directional pattern suggesting bias was observed during visual inspection of the available data, publication-bias evaluation remains inherently constrained by the small number of contributing studies.

## 4 Discussion

Transcatheter edge-to-edge repair is a percutaneous procedure designed to reduce mitral regurgitation. To our knowledge, this is the first meta-analysis in heart failure patients with secondary mitral regurgitation since the COAPT (13), MITRA-FR (12) and Reshape-HF2 (29) trials to comprehensively investigate echocardiographic parameters to assess reverse remodeling, hypothesizing that the beneficial effects of TEER might be mediated through this mechanism (5). Although cardiac remodeling predicts a poor prognosis, its reversal has been associated with improved survival. For instance, one study reported a 3% mortality rate at 17 months in patients with reverse cardiac remodeling compared to 22% in patients without such changes (30). This can be effectively achieved through coronary revascularization, optimal medical therapy such as renin–angiotensin–aldosterone system inhibitors (31), β-blockers (4, 32), mineralocorticoid receptor antagonists, SGLT-2 inhibitors and device therapies such as cardiac resynchronization therapy (33) or ventricular assist devices (34, 35). In isolated SMR surgical intervention is limited due to significant procedural risks, high rates of recurrent MR, and lack of proven survival benefits (11, 36, 37).

### 4.1 Patient selection

Three large RCTs, the COAPT (13), MITRA-FR (12), and Reshape-HF2 (29) trials, evaluated the safety and efficacy of TEER in patients with symptomatic HF and severe SMR. COAPT demonstrated that TEER significantly reduced hospitalizations for heart failure and all-cause mortality compared to GDMT alone, whereas the recently published Reshape-HF2 showed lower rates of first or recurrent hospitalizations for heart failure or cardiovascular mortality compared to GDMT alone. In contrast, MITRA-FR found no significant impact on mortality or heart failure hospitalizations (38).

The conflicting results between these trials may be due to differences in patient selection and trial design, echocardiographic methodology and follow-up and the use of GDMT. Multiple studies have shown that extensive LV dilation (LV end-diastolic diameter >65 mm) and LV dysfunction (LVEF <20%, LV end-systolic diameter >55 mm) are associated with less reverse LV remodeling (39). Differences in baseline characteristics between RCTs highlight this issue. In the COAPT and Reshape-HF2 trials, patients had more severe SMR, had smaller LV end-diastolic volumes and higher LVEF compared to those in the MITRA-FR trial that included patients with less severe SMR and more significant LV dilation. These differences highlight the importance of correct patient selection for optimal results with the TEER procedure (39). According to the 2025 ESC guidelines on valvular heart disease TEER is recommended in symptomatic ventricular SMR patients with specific clinical and echocardiographic criteria. In patients with advanced heart failure (when not suitable for LVAD or heart transplantation), or not entirely fulfilling all criteria or with atrial SMR TEER may be considered. to improve symptoms, functional capacity and quality of life (40).

### 4.2 Baseline to follow-up changes

In accordance with other authors, we defined clinically relevant reverse remodeling as a minimum 10% improvement in echocardiographic parameters (41). Although most echocardiographic parameter changes were statistically significant, thus favouring TEER, such as LVEDD (−1.63 mm), LVESD (−1.20 mm), LVEDV (−14.21 mL), LVESV (−9.24 mL), and LAVi (−5.70 mL), their clinical relevance is questionable, none of these changes reached the criteria of reverse remodeling.

We observed that the most significant changes occurred during the 6-month follow-up. During this period, changes in LVEDD (−1.94 mm), LVESD (−2.39 mm), LVEDV (−21.09 mL), and LVESV (−13.25 mL) were more pronounced compared to other follow-up periods. We believe that the potential favorable effects of TEER on cardiac remodeling may occur at some latency and diminish after one year, given that heart failure is a progressive disease. Although some meta-analyses reported significant improvements in parameters such as LVEF, LVESV, and LVEDV, others, including our own, did not find such marked changes. For example, D'Ascenzo et al. reported improvements in LVEF (4%), LVESV (−22 mL), and LVEDV (−25 mL) (42), whereas Megaly et al. found reductions in LVEDV (−14.24 mL), LVESV (−7.67 mL), LVEDD (−2.92 mm), and LVESD (−1.92 mm) (43). The variation in findings across studies may be due to differences in study populations, methodologies, follow-up durations, and baseline patient characteristics, such as the severity of heart failure and mitral regurgitation.

### 4.3 GDMT vs. TEER + GDMT effect

Although TEER effectively reduced MR severity, it did not demonstrate superiority over GDMT alone in promoting reverse remodeling. This finding likely reflects a combination of clinical and mechanistic factors. First, many patients undergoing TEER had advanced LV dilation or impaired contractile reserve, where the myocardium has limited ability to recover structural geometry despite reduced regurgitant volume. Second, reverse remodeling is a progressive process that may require longer follow-up than reported by most cohorts (typically ≤12 months). Third, contemporary GDMT—including ARNI, β-blockers, MRAs, and SGLT-2 inhibitors, frequently combined with CRT—can independently induce reverse remodeling, thereby reducing the measurable incremental effect of TEER. Lastly, TEER targets the regurgitant mechanism but does not modify the cardiomyopathic substrate that drives disease progression, which may explain the observed dissociation between improved hemodynamics (e.g., NT-proBNP reduction) and limited structural response. The neutral comparison between TEER + GDMT and GDMT alone likely reflects the underlying pathophysiology of functional MR rather than insufficient procedural efficacy. In patients with severely dilated ventricles, markedly elevated (indexed) LVEDV, or long-standing cardiomyopathic remodeling, the myocardium often has limited capacity for structural recovery even when regurgitant volume is reduced. Conversely, patients with more favorable ventricular geometry and preserved right ventricular function may retain a greater potential for reverse remodeling. Differences in patient selection between cohorts resembling COAPT (less dilation, fewer concomitant right-sided abnormalities, more “proportionate” MR) and MITRA-FR (larger ventricles, more advanced disease, higher rates of RV dysfunction and TR) likely contributed to the heterogeneity observed across studies. These mechanistic considerations suggest that TEER's structural impact is constrained by the underlying myocardial substrate, and that ventricular geometry—not only MR reduction—plays a central role in determining responsiveness to therapy.

### 4.4 Technical success

The primary goal of the operation was achieved with great success, because the proportion of patients with grade 3–4 mitral regurgitation was reduced from 99% to 9% after the procedure. This underscores the efficacy of the TEER procedure in reducing the severity of mitral regurgitation.

### 4.5 NT-proBNP

However only a few studies reported NT-proBNP levels, but they were decreased by almost half, due to reduced wall stretching and improved hemodynamic functions. This significant reduction in NT-proBNP levels indicates that the TEER procedure helps alleviate the burden on the cardiac muscle, resulting in a positive impact on overall cardiac function and symptom relief for patients.

### 4.6 Strengths and limitations

Our study has a unique design that, to the best of our knowledge, has not been previously used in meta-analyses on this topic, providing new insights. The relatively large number of patients across the studies included increases the impact and generalizability of our analysis. In addition, we examined different follow-up periods, allowing for a more detailed assessment of the effects of the TEER procedure over various timeframes. Finally, we rigorously adhered to all Cochrane Collaboration guidelines, ensuring the highest level of quality, transparency, and reproducibility of the results (44).

However, several important limitations must be acknowledged. First, as this meta-analysis is based predominantly on observational studies, the certainty of evidence was rated as low according to the GRADE framework (27). Although several included studies were judged to have high risk of bias, we did not observe a consistent directional distortion of effect estimates; rather, these limitations introduce random uncertainty into the results. Second, substantial between-study heterogeneity was present for multiple outcomes, driven by variations in sample size, effect size, and confidence interval widths, which further reduces confidence in the pooled estimates. Third, reporting across studies was inconsistent—particularly regarding MR severity, GDMT optimization, CRT use, and other clinically relevant characteristics—which prevented meaningful subgroup analyses or meta-regression despite reviewer suggestions. Fourth, publication bias could not be reliably assessed for most outcomes, as fewer than ten studies contributed data per endpoint, limiting interpretability of funnel plots. Finally, the evidence base remains constrained by the lack of prospective randomized trials directly comparing TEER with GDMT; therefore, the clinical implications of the observed statistically significant effects should be interpreted with caution. Future research should focus on standardized reporting, phenotype-specific analyses, and adequately powered randomized studies to better define which patient populations derive the greatest benefit from TEER.

## 5 Conclusion

While TEER effectively reduces mitral regurgitation severity, its impact on left ventricular reverse remodeling appears limited and inconsistent across studies. Considering the high heterogeneity and low certainty of available evidence, these findings should be interpreted cautiously, and carefully designed future studies are needed to clarify which patient subgroups may benefit most.

## Data Availability

The original contributions presented in the study are included in the article/Supplementary Material, further inquiries can be directed to the corresponding authors.
