# Supplementary material for: Assessing the impact of transcatheter edge-to-edge repair on reverse remodeling in secondary mitral regurgitation: a systematic review and meta-analysis
Source: Front Cardiovasc Med. 2026 Jan 30;12:1714337. doi: 10.3389/fcvm.2025.1714337 (PMC12901449; doi:10.3389/fcvm.2025.1714337)
Supplement: Supplementary file 1 [file Datasheet1.docx]

# Supplementary Material

**Title:**

Assessing the Impact of Transcatheter Edge-to-Edge Repair on Reverse Remodeling in Secondary Mitral Regurgitation: A Systematic Review and Meta-Analysis

**Authors**

Adolf Lichtfusz^1,2^, Nina Galdzytska^1^, Dorottya Gergő^1,3^, Bence Szabó^1^, Péter Hegyi^1,4,5^, Zsolt Molnár^1,6,7^, Gábor Duray^1,2,8^, Judit Papp^1,2^.

**Affiliations:**

1. Centre for Translational Medicine, Semmelweis University, Budapest, Hungary
2. Department of Cardiology, Central Hospital of Northern Pest - Military Hospital, Budapest, Hungary
3. Department of Pharmacognosy, Semmelweis University, Budapest, Hungary
4. Institute for Translational Medicine, Medical School, University of Pécs, Pécs, Hungary
5. Institute of Pancreatic Diseases, Semmelweis University, Budapest, Hungary
6. Department of Anesthesiology and Intensive Therapy, Semmelweis University, Budapest, Hungary
7. Department of Anesthesiology and Intensive Therapy, Poznan University of Medical Sciences, Poznan, Poland
8. Semmelweis University, Heart and Vascular Center, Budapest, Hungary

**Table of contents:**

**Figure S1. Effect of TEER+GDMT on left ventricular end-systolic diameter – 1 month, 6 months, 12 months, and mixed-length follow-up.**

**Figure S2. Effect of TEER+GDMT on indexed left ventricular end-diastolic volume – 1 month, 6 months, 12 months, and mixed-length follow-up.**

**Figure S3. Effect of TEER+GDMT on left ventricular end-systolic volume – 1 month, 6 months, 12 months, and mixed-length follow-up.**

**Figure S4. Effect of TEER+GDMT on left atrial volume – 1 month, 6 months, 12 months, and mixed-length follow-up.**

**Figure S5. Effect of TEER+GDMT on indexed left atrial volume – 1 month, 6 months, 12 months, and mixed-length follow-up.**

**Figure S6a. Proportion of grade 3 mitral regurgitation before TEER– 1 month, 6 months, 12 months, and mixed follow-up length.**

**Figure S6b. Proportion of grade 3 mitral regurgitation after TEER– 1 month, 6 months, 12 months, and mixed follow-up length.**

**Figure S7. Effect of TEER+GDMT compared to GDMT alone on ejection fraction.**

**Figure S8. Effect of TEER+GDMT compared to GDMT alone on left ventricular end-diastolic volume.**

**Figure S9. Effect of TEER+GDMT compared to GDMT alone on left ventricular end-systolic volume.**

**Table S1. PRISMA checklist**

**Table S2. GRADE: TEER + GDMT compared to GDMT alone for reverse remodelling**

**Table S3. GRADE: TEER + GDMT effect on reverse remodelling over time.**

**Table S4. Search key and settings for different databases**

**Table S5 – Additional baseline characteristics studies comparing TEER+GDMT vs GDMT alone**

**Table S6 – Additional baseline characteristics studies investigating TEER+GDMT effect over time**

**S2.5 Statistical analysis**

**Figure S1. Effect of TEER+GDMT on left ventricular end-systolic diameter – 1 month, 6 months, 12 months, and mixed-length follow-up.**

**Figure S2. Effect of TEER+GDMT on indexed left ventricular end-diastolic volume – 1 month, 6 months, 12 months, and mixed-length follow-up.**

**Figure S3. Effect of TEER+GDMT on left ventricular end-systolic volume – 1 month, 6 months, 12 months, and mixed-length follow-up.**

**Figure S4. Effect of TEER+GDMT on left atrial volume – 1 month, 6 months, 12 months, and mixed-length follow-up.**

**Figure S5. Effect of TEER+GDMT on indexed left atrial volume – 1 month, 6 months, 12 months, and mixed-length follow-up.**

**Figure S6a. Proportion of grade 3 mitral regurgitation before TEER– 1 month, 6 months, 12 months, and mixed follow-up follow-up length.**

**Figure S6b. Proportion of grade 3 mitral regurgitation after TEER– 1 month, 6 months, 12 months, and mixed follow-up follow-up length.**

**Figure S7. Effect of TEER+GDMT compared to GDMT alone on ejection fraction. MD-mean difference, CI-confidence interval, SD-standard deviation.**

**Figure S8. Effect of TEER+GDMT compared to GDMT alone on left ventricular end-diastolic volume.**

**Figure S9. Effect of TEER+GDMT compared to GDMT alone on left ventricular end-systolic volume.**

**Table S1. PRISMA checklist**


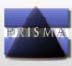
**PRISMA 2020 Checklist**

| **Section and Topic** | **Item #** | **Checklist item** | **Location where item is reported** |
| --- | --- | --- | --- |
| **TITLE** | | |  |
| Title | 1 | Identify the report as a systematic review. | Title |
| **ABSTRACT** | | |  |
| Abstract | 2 | See the PRISMA 2020 for Abstracts checklist. | Abstract |
| **INTRODUCTION** | | |  |
| Rationale | 3 | Describe the rationale for the review in the context of existing knowledge. | Introduction |
| Objectives | 4 | Provide an explicit statement of the objective(s) or question(s) the review addresses. | Last sentence from the introduction |
| **METHODS** | | |  |
| Eligibility criteria | 5 | Specify the inclusion and exclusion criteria for the review and how studies were grouped for the syntheses. | 2.3 |
| Information sources | 6 | Specify all databases, registers, websites, organisations, reference lists and other sources searched or consulted to identify studies. Specify the date when each source was last searched or consulted. | 2.2 |
| Search strategy | 7 | Present the full search strategies for all databases, registers and websites, including any filters and limits used. | 2.2 |
| Selection process | 8 | Specify the methods used to decide whether a study met the inclusion criteria of the review, including how many reviewers screened each record and each report retrieved, whether they worked independently, and if applicable, details of automation tools used in the process. | 2.4 |
| Data collection process | 9 | Specify the methods used to collect data from reports, including how many reviewers collected data from each report, whether they worked independently, any processes for obtaining or confirming data from study investigators, and if applicable, details of automation tools used in the process. | 2.4 |
| Data items | 10a | List and define all outcomes for which data were sought. Specify whether all results that were compatible with each outcome domain in each study were sought (e.g. for all measures, time points, analyses), and if not, the methods used to decide which results to collect. | 2.4 |
|  | 10b | List and define all other variables for which data were sought (e.g. participant and intervention characteristics, funding sources). Describe any assumptions made about any missing or unclear information. | 2.4 |
| Study risk of bias assessment | 11 | Specify the methods used to assess risk of bias in the included studies, including details of the tool(s) used, how many reviewers assessed each study and whether they worked independently, and if applicable, details of automation tools used in the process. | 2.5 |
| Effect measures | 12 | Specify for each outcome the effect measure(s) (e.g. risk ratio, mean difference) used in the synthesis or presentation of results. | 2.5 |
| Synthesis methods | 13a | Describe the processes used to decide which studies were eligible for each synthesis (e.g. tabulating the study intervention characteristics and comparing against the planned groups for each synthesis (item #5)). | 2.5 |
|  | 13b | Describe any methods required to prepare the data for presentation or synthesis, such as handling of missing summary statistics, or data conversions. | 2.5 |
|  | 13c | Describe any methods used to tabulate or visually display results of individual studies and syntheses. | 2.5 |
|  | 13d | Describe any methods used to synthesize results and provide a rationale for the choice(s). If meta-analysis was performed, describe the model(s), method(s) to identify the presence and extent of statistical heterogeneity, and software package(s) used. | 2.5 |
|  | 13e | Describe any methods used to explore possible causes of heterogeneity among study results (e.g. subgroup analysis, meta-regression). | 2.5 |
|  | 13f | Describe any sensitivity analyses conducted to assess robustness of the synthesized results. | 2.5 |
| Reporting bias assessment | 14 | Describe any methods used to assess risk of bias due to missing results in a synthesis (arising from reporting biases). | 2.6 |
| Certainty assessment | 15 | Describe any methods used to assess certainty (or confidence) in the body of evidence for an outcome. | 2.7 |
| **RESULTS** | | |  |
| Study selection | 16a | Describe the results of the search and selection process, from the number of records identified in the search to the number of studies included in the review, ideally using a flow diagram. | 3.1 |
|  | 16b | Cite studies that might appear to meet the inclusion criteria, but which were excluded, and explain why they were excluded. | 3.1 |
| Study characteristics | 17 | Cite each included study and present its characteristics. | 3.2 |
| Risk of bias in studies | 18 | Present assessments of risk of bias for each included study. | Plots |
| Results of individual studies | 19 | For all outcomes, present, for each study: (a) summary statistics for each group (where appropriate) and (b) an effect estimate and its precision (e.g. confidence/credible interval), ideally using structured tables or plots. | Plots |
| Results of syntheses | 20a | For each synthesis, briefly summarise the characteristics and risk of bias among contributing studies. | Plots |
|  | 20b | Present results of all statistical syntheses conducted. If meta-analysis was done, present for each the summary estimate and its precision (e.g. confidence/credible interval) and measures of statistical heterogeneity. If comparing groups, describe the direction of the effect. | Plots |
|  | 20c | Present results of all investigations of possible causes of heterogeneity among study results. | 3.2 |
|  | 20d | Present results of all sensitivity analyses conducted to assess the robustness of the synthesized results. | 2.5 |
| Reporting biases | 21 | Present assessments of risk of bias due to missing results (arising from reporting biases) for each synthesis assessed. | Plots, corresponding domain |
| Certainty of evidence | 22 | Present assessments of certainty (or confidence) in the body of evidence for each outcome assessed. | GRADE on the figure, Supplementary material |
| **DISCUSSION** | | |  |
| Discussion | 23a | Provide a general interpretation of the results in the context of other evidence. | 4.1 |
|  | 23b | Discuss any limitations of the evidence included in the review. | 4.6 |
|  | 23c | Discuss any limitations of the review processes used. | 4.6 |
|  | 23d | Discuss implications of the results for practice, policy, and future research. | 4.7 |
| **OTHER INFORMATION** | | |  |
| Registration and protocol | 24a | Provide registration information for the review, including register name and registration number, or state that the review was not registered. | 2.2 |
|  | 24b | Indicate where the review protocol can be accessed, or state that a protocol was not prepared. | 2.2 |
|  | 24c | Describe and explain any amendments to information provided at registration or in the protocol. | 2.2 |
| Support | 25 | Describe sources of financial or non-financial support for the review, and the role of the funders or sponsors in the review. | 6.2 |
| Competing interests | 26 | Declare any competing interests of review authors. | 6.3 |
| Availability of data, code and other materials | 27 | Report which of the following are publicly available and where they can be found: template data collection forms; data extracted from included studies; data used for all analyses; analytic code; any other materials used in the review. | Table S4 |

**Table S2. GRADE: TEER + GDMT compared to GDMT alone for reverse remodeling**

Grade Assessment

| **Certainty assessment** | | | | | | | **№ of patients** | | **Effect** | | **Certainty** | **Importance** |
| --- | --- | --- | --- | --- | --- | --- | --- | --- | --- | --- | --- | --- |
| **№ of studies** | **Study design** | **Risk of bias** | **Inconsistency** | **Indirectness** | **Imprecision** | **Other considerations** | **TEER** | **GDMT alone** | **Relative (95% CI)** | **Absolute (95% CI)** |  |  |
| **Left ventricular end diastolic diameter (follow-up: range 6 months to 12 months; assessed with: MM)** | | | | | | | | | | | | |
| 4 | non-randomised studies | not serious | not serious | very serious | not serious | none | 435 | 426 | - | MD **1.02 MM lower** (2.81 lower to 0.78 higher) | ⨁⨁◯◯ Low | IMPORTANT |
| **Ejection Fraction (follow-up: range 6 months to 12 months; assessed with: %)** | | | | | | | | | | | | |
| 6 | non-randomised studies | not serious | serious | not serious | serious | none | 480 | 449 | - | MD **0.14 % lower** (3.07 lower to 2.79 higher) | ⨁⨁◯◯ Low | IMPORTANT |
| **Left ventricular end diastolic volume raw (follow-up: mean 12 months)** | | | | | | | | | | | | |
| 3 | non-randomised studies | not serious | serious | serious | not serious | none | 428 | 403 | - | MD **7.93 lower** (41.8 lower to 25.95 higher) | ⨁⨁◯◯ Low | IMPORTANT |
| **Left ventricular end systolic volume raw (follow-up: mean 12 months; assessed with: ml)** | | | | | | | | | | | | |
| 3 | non-randomised studies | not serious | not serious | very serious | not serious | none | 425 | 399 | - | MD **5.29 lower** (28.55 lower to 17.98 higher) | ⨁⨁◯◯ Low | IMPORTANT |

**CI:** confidence interval; **MD:** mean difference

**Question 1:** TEER + GDMT compared to GDMT alone for reverse remodeling

**Table S3. GRADE: TEER + GDMT effect on reverse remodeling over time.**

| **№ of studies** | **Certainty assessment** | | | | | | **Effect** | | | **Certainty** | **Importance** |
| --- | --- | --- | --- | --- | --- | --- | --- | --- | --- | --- | --- |
|  | **Study design** | **Risk of bias** | **Inconsistency** | **Indirectness** | **Imprecision** | **Other considerations** | **№ of events** | **№ of individuals** | **Rate (95% CI)** |  |  |
| Left ventricular end diastolic diameter (assessed with: mm) | | | | | | | | | | | |
| 14 | non-randomised studies | serious | serious | serious | serious | none | - | 2249 | MD -1.63 (-2.41 to -0.85) | ⨁◯◯◯ Very low | IMPORTANT |
| Left ventricular end diastolic volume (assessed with: ml) | | | | | | | | | | | |
| 16 | non-randomised studies | serious | serious | not serious | not serious | none | - | 1907 | MD -14.21 (-19.42 to -9) | ⨁⨁◯◯ Low | IMPORTANT |
| Left atrial volume (assessed with: ml) | | | | | | | | | | | |
| 8 | non-randomised studies | serious | very serious | very serious | serious | none | - | 1.002 | MD -5.70 (-15.75 to 4.35) | ⨁◯◯◯ Very low | IMPORTANT |
| Left ventricular end systolic diameter (assessed with: mm) | | | | | | | | | | | |
| 16 | non-randomised studies | serious | not serious | serious | not serious | none | - | 2201 | MD -1.20 (-1.97 to -0.43) | ⨁⨁◯◯ Low | IMPORTANT |
| Left atrial volume indexed (assessed with: ml/m2) | | | | | | | | | | | |
| 9 | non-randomised studies | serious | very serious | serious | serious | none | - | 986 | MD -6.86 (-12.79 to -0.94) | ⨁◯◯◯ Very low | IMPORTANT |
| Left ventricular end diastolic volume - indexed (assessed with: ml) | | | | | | | | | | | |
| 9 | non-randomised studies | serious | very serious | serious | serious | none | - | 344 | MD -4.08 (-10.26 to 2.1) | ⨁◯◯◯ Very low | IMPORTANT |
| Left ventricular end systolic volume - raw (assessed with: ml) | | | | | | | | | | | |
| 19 | non-randomised studies | very serious | very serious | serious | not serious | none | - | 2068 | MD -9.24 (-14 to -4.48) | ⨁◯◯◯ Very low | IMPORTANT |
| NT-ProBNP (assessed with: pg/mL) | | | | | | | | | | | |
| 4 | non-randomised studies | serious | serious | not serious | not serious | none | - | 237 | MD -1340.21 (-2197.63 to -482.79) | ⨁⨁◯◯ Low | IMPORTANT |
| Ejection Fraction (assessed with: %) | | | | | | | | | | | |
| 29 | non-randomised studies | very serious | very serious | serious | not serious | none | - | 2471 | MD 1.10 (0.06 to 2.14) | ⨁◯◯◯ Very low | IMPORTANT |

**Question 2 :** TEER + GDMT effect on reverse remodeling overtime.

**Table S4. Searchkey and settings for different databases**

| **Searchkey** |
| --- |
| **("mitral" AND ("valve" OR regurg* OR insuf*) AND ("moderate" OR "severe"))   AND**  **(percutan* or mitraclip* or pascal* or ("transcatheter" AND "edge-to-edge" AND repair*) OR ("transcatheter" AND "mitral" AND "valve" AND repair*) or "teer" or "tmvr")**  1st domain: Patient characteristics 2nd domain: Intervention technique |
| Pubmed: 2569 results  No year or study type restriction was used. |
| EMBASE: 6418 results We used the advanced search and unchecked every checkmark. With these settings, there was no explosion for any words. |
| Cochrane Library: 303 results for Trials We used the advanced search setting up ‘All Text’  We did not use any quotation marks as the Cochrane Search assumes “AND” between the words of multi-word phrases.  (mitral AND (valve OR regurg* OR insuf*) AND (moderate OR severe)) AND (percutan* or mitraclip* or pascal* or (transcatheter edge-to-edge repair*) OR (transcatheter AND mitral AND valve AND repair*) or teer or tmvr) |
| **In total we got 9290 hits.** |

Table S5 – Additional baseline characteristics **studies comparing TEER+GDMT vs GDMT alone**

| \| **First author (year)** \| **Baseline MR severity (EROA)** \| **Regurgitant volume (mL)** \| **β-blocker (%)** \| **ACEi/ARB/ARNI (%)** \| **MRA (%)** \| **SGLT2i (%)** \| **CRT (%)** \| **Baseline NYHA (short form)** \| **RV dysfunction / TR** \| \| --- \| --- \| --- \| --- \| --- \| --- \| --- \| --- \| --- \| --- \| \| Asch et al. 2019 (COAPT echo substudy) \| 0.41 ± 0.15 cm² \| NR \| NR \| NR \| NR \| NR \| NR \| NR \| TR ≥2+ in 16.4% at baseline \| \| Freixa et al. 2022 (MITRA-CRT) \| 0.51 ± 0.12 cm² \| NR \| 94 \| ACEi/ARB 35; ARNI 32 \| 81 \| NR \| 100 (all had CRT with >95% BiV pacing) \| NYHA II 13, III 81, IV 7 (overall; TEER 6/81/13 vs control 20/80/0) \| NR \| \| Hubert et al. 2019 (MITRA-FR echo analysis) \| 31 ± 11 mm² \| 45 ± 14 (both groups) \| NR \| NR \| NR \| NR \| NR \| NYHA II–IV; mean NYHA 2.5 ± 0.5 (TEER 2.6, control 2.3) \| NR \| \| Krawczyk-Ożóg et al. 2018 \| TEER: 25.1 ± 7.7 mm²; Control: 26.1 ± 7.3 mm² \| TEER: 37.6 ± 13.8; Control: 38.4 ± 9.4 \| NR \| NR \| NR \| NR \| TEER: 70; Control: 26.1 \| TEER: II 10, III 70, IV 20; Control: II 8.7, III 73.9, IV 17.4 \| Severe TR TEER: 0% baseline, 12.5% FU; Control: 21.1% baseline, 26.3% FU \| \| Obadia et al. 2018 (MITRA-FR main trial) \| 31 ± 10 mm² (both groups) \| TEER: 45 ± 13, Control: 45 ± 14 \| 91.4 \| ACEi/ARB/ARNI 91.4 (TEER), 92.1 (control) \| MRA 65.8 (TEER), 66.4 (control) \| NR \| TEER: 20.4, Control 19.1 \| TEER: II 36.8, III 53.9, IV 9.2; Control: II 28.9, III 63.2, IV 7.9 \| NR \| \| Papadopoulos et al. 2020 \| 28.9 ± 13.9 mm² \| 42.9 ± 17.3 \| 96.4 \| ACEi 89.2 \| MRA 92.8 \| NR \| 34.9 \| Mean NYHA 3.1 ± 0.6 (majority III–IV) \| NR \| |
| --- | --- | --- | --- | --- | --- | --- | --- | --- | --- | --- | --- | --- | --- | --- | --- | --- | --- | --- | --- | --- | --- | --- | --- | --- | --- | --- | --- | --- | --- | --- | --- | --- | --- | --- | --- | --- | --- | --- | --- | --- | --- | --- | --- | --- | --- | --- | --- | --- | --- | --- | --- | --- | --- | --- | --- | --- | --- | --- | --- | --- | --- | --- | --- | --- | --- | --- | --- | --- | --- | --- |

**Table S6 – Additional baseline characteristics studies investigating TEER+GDMT effect over time**

| \| **First author (year / subgroup)** \| **Baseline MR severity (EROA)** \| **Regurgitant volume (mL)** \| **β-blocker (%)** \| **ACEi/ARB/ARNI (%)** \| **MRA (%)** \| **SGLT2i (%)** \| **CRT (%)** \| **Baseline NYHA (short form)** \| **RV dysfunction / TR** \| \| --- \| --- \| --- \| --- \| --- \| --- \| --- \| --- \| --- \| --- \| \| Ailawadi et al. 2019 \| NR \| NR \| 79.7 \| ACEi/ARB 63.3 \| NR \| NR \| NR \| I 2.1, II 17.4, III 62.9, IV 17.6 \| NR \| \| Albini et al. 2022 \| NR \| NR \| 87.5 \| ACEi 16.6, ARB 33.3 \| 91.6 \| NR \| NR \| III 70.8, IV 29.2 \| Reduced TAPSE 14.0 mm (RV dysfunction); TR Vmax 2.9 m/s (significant TR) \| \| Altiok et al. 2012 \| 0.33 ± 0.15 cm² \| 84.1 ± 38.3 \| NR \| NR \| NR \| NR \| NR \| III 69, IV 31 \| NR \| \| Barth et al. 2017 (LVEF <20%) \| 0.41 ± 0.15 cm² \| NR \| NR \| NR \| NR \| NR \| CRT 42 \| NYHA III–IV 83 \| NR \| \| Barth et al. 2017 (LVEF ≥20%) \| 0.37 ± 0.14 cm² \| NR \| NR \| NR \| NR \| NR \| CRT 18 \| NYHA III–IV 92 \| NR \| \| Barth et al. 2021 (PASCAL) \| EROA overall 0.43 ± 0.2 cm² (FMR 0.39, DMR 0.51) \| NR \| NR \| NR \| NR \| NR \| CRT 4.3 overall (6.3 FMR, 0 DMR) \| NYHA III–IV ≈99 (100% FMR, 96.6% DMR) \| Significant TR excluded (TR was an exclusion criterion) \| \| Berardini et al. 2017 \| NR \| NR \| 87.5 \| ACEi 16.6, ARB 33.3 \| 91.6 \| NR \| CRT-D 44 \| III 80, IV 20 \| RV dysfunction ≥ moderate 39%; TR grade not quantified \| \| Buck et al. 2021 \| NR \| NR \| NR \| NR \| NR \| NR \| NR \| Mean NYHA ≈3.1 (FMR) and 3.0 (DMR); class distribution NR \| NR \| \| Chan et al. 2012 \| NR \| NR \| NR \| NR \| NR \| NR \| NR \| Mean NYHA 3.1 (FMR), 3.0 (DMR); distribution NR \| NR \| \| Cimino et al. 2019 \| 0.37 ± 0.15 cm² \| 51 ± 14.3 \| NR \| NR \| NR \| NR \| NR \| 100% NYHA III–IV \| Non-LVRR group with larger RV end-systolic area; TR not explicitly quantified \| \| Citro et al. 2017 \| NR \| NR \| 87.8 \| ACEi/ARB 68.3 \| 48.8 \| NR \| 9.8 \| II 7.3, III 85.4, IV 7.3 \| RV function improved (TAPSE ↑, PASP ↓); significant TR not quantified \| \| Demir et al. 2020 \| NR \| NR \| NR \| NR \| NR \| NR \| NR \| NYHA improvement described; exact baseline class distribution NR \| NR \| \| El Shurafa et al. 2021 \| NR \| NR \| 90.9 (FMR), 78.3 (DMR) \| ACEi/ARB 76.1 (FMR), 56.5 (DMR) \| MRA 54.6 (FMR), 13.0 (DMR) \| NR \| NR \| NYHA III/IV 96.6 (FMR), 86.9 (DMR) \| NR \| \| Giaimo et al. 2018 \| NR \| NR \| 96.7 \| ACEi/ARB 73.3 \| 66.7 \| NR \| CRT-D 90, CRT-P 10 \| III 83.3, IV 16.7 \| NR \| \| Giannini et al. 2014 \| NR \| NR \| NR \| NR \| NR \| NR \| NR \| NYHA III/IV 68.5 (rest II) \| RV dysfunction reported as prognostic; TR not quantified \| \| Godino et al. 2016 \| NR \| NR \| 78 \| ACEi/ARB 52 \| 48 \| NR \| CRT-D 18, CRT-PM 17 \| II 30, III 60, IV 10 \| RV dysfunction 37%; TR >2 in 25% \| \| González et al. 2020 (Benito-González) \| NR \| NR \| 89.3 \| ACEi/ARB/ARNI 86.2 \| 74.2 \| NR \| CRT (±ICD) 43.0 \| II 23.7, III 65.6, IV 10.8 \| NR \| \| Hagnäs et al. 2021 (decreased LVEF) \| NR \| NR \| NR \| – \| – \| – \| CRT pacemaker 10 \| II 14, III 74, IV 12 \| NR \| \| Hagnäs et al. 2021 (improved LVEF) \| NR \| NR \| NR \| – \| – \| – \| CRT pacemaker 10 \| II 14, III 74, IV 12 \| NR \| \| Hagnäs et al. 2021 (unchanged LVEF) \| NR \| NR \| NR \| – \| – \| – \| CRT pacemaker 10 \| II 14, III 74, IV 12 \| NR \| \| Han Yoon et al. 2022 (a-FMR) \| 0.30 cm² \| NR \| NR \| NR \| NR \| NR \| Prior CRT 33.6 \| NYHA III/IV 94.8 \| Moderate/severe TR 54.3; severe TR 21.6 \| \| Han Yoon et al. 2022 (v-FMR) \| 0.35 cm² \| NR \| NR \| NR \| NR \| NR \| Prior CRT 50.7 \| NYHA III/IV 95.0 \| Moderate/severe TR 49.7; severe TR 16.2 \| \| Kamperidis et al. 2018 \| 0.21 ± 0.10 cm² \| 31.5 ± 12.3 \| 69 \| ACEi/ARB 82 \| NR \| NR \| 26 \| NYHA III–IV 63 \| NR \| \| Nickenig et al. 2014 \| 0.43 cm² overall (0.42 FMR, 0.46 DMR) \| 53.8 overall (51.1 FMR, 62.7 DMR) \| NR \| NR \| NR \| NR \| NR \| I 1.6, II 12.9, III 68.7, IV 16.8 \| NR \| \| Nita et al. 2020 (LVRR) \| NR \| NR \| NR \| NR \| NR \| NR \| 11.1 \| NYHA I 6.2, II 8.6, III 59.3, IV 25.9 \| Severe TR baseline 49.4; 12-month 19.8 \| \| Nita et al. 2020 (no LVRR) \| NR \| NR \| NR \| NR \| NR \| NR \| 9.6 \| NYHA I 3.6, II 9.6, III 61.5, IV 25.3 \| Severe TR baseline 45.8; 12-month 32.5 \| \| Ohno et al. 2014 (Moderate/Severe TR) \| Severe MR: EROA >40 mm² or RV >60 \| TR severe: EROA ≥40 or RV ≥45 \| NR \| NR \| NR \| NR \| NR \| NYHA II 10.6, III 70.2, IV 19.1 \| Moderate/severe TR group (32.2% overall had moderate/severe TR); RV dysfunction NR \| \| Ohno et al. 2014 (None/Mild TR) \| NR \| – \| NR \| NR \| NR \| NR \| NR \| NYHA II 23.2, III 70.7, IV 6.1 \| None/mild TR by definition \| \| Orban et al. 2023 (EXPAND) \| 0.30 ± 0.12 cm² \| 44.8 ± 19.4 \| 88.6 \| ACEi 37.8, ARB 22.3, ARNI 11.6 \| 36.6 \| NR \| 10.2 \| I 1.2, II 15.7, III 69.5, IV 13.6 \| TR ≥2+ in 42%; RV dysfunction NR \| \| Öztürk et al. 2021 \| 0.30 ± 0.06 cm² \| 51.4 ± 13.4 per beat \| 80 \| ACEi/ARB 66 \| NR \| NR \| 8 \| 100% NYHA >II (III–IV) \| Mean TR grade 2.1 ± 0.2; RV dysfunction not explicitly reported \| \| Palmiero et al. 2017 \| Mild MR: EROA 0.14 ± 0.08; Moderate: 0.21 ± 0.11; Severe: 0.29 ± 0.08 \| NR \| NR \| NR \| NR \| NR \| NR \| Mean NYHA: mild 1.6, moderate 2.9, severe 3.2; NYHA III–IV 46% / 60% / 96% \| NR \| \| Perl et al. 2013 \| NR \| NR \| NR \| NR \| NR \| NR \| NR \| Mean NYHA 3.4 ± 0.5 (distribution NR) \| NR \| \| Scandura et al. 2012 \| NR (quantified but values not in text) \| NR \| NR \| NR \| NR \| NR \| 11.4 \| II 15.9, III 68.2, IV 15.9 \| NR \| \| Shechter et al. 2023 \| 0.36 cm² (median, IQR 0.26–0.46) \| 44.5 mL (median, IQR 30.7–61.1) \| 82.3 \| ACEi/ARB 36.4, ARNI 15.6 \| 42.7 \| 1.1 \| CRT/ICD50 \| II 2.1, III 31.3, IV 66.7 \| RV dysfunction 86.7% (moderate/severe 53.3%); TR >moderate 35.4% \| \| Taramasso et al. 2012 (MitraClip arm) \| NR \| NR \| NR \| NR \| NR \| NR \| NR \| NYHA II 15.4, III 63.3, IV 17.3 \| Significant TR 23.6% (vs 17.1% surgery group) \| \| Tay et al. 2015 (Asia-Pacific registry) \| NR \| NR \| NR \| NR \| NR \| NR \| NR \| I 1.2, II 28.2, III 57.1, IV 13.5 \| TR grade 2+ decreased in FMR and DMR (e.g. FMR 21.6%→10.7% at 30 days); RV dysfunction NR \| \| Toprak et al. 2016 \| NR \| NR \| NR \| NR \| NR \| NR \| NR \| Baseline NYHA not specified in detail \| NR \| \| Vitarelli et al. 2015 \| 33.1 ± 13.8 mm² \| 44.2 ± 11.4 \| NR \| NR \| NR \| NR \| 9.4 \| NYHA III/IV 90.6; mean NYHA 3.2 ± 0.6 \| Severe RV dysfunction (3D RVEF <40%, RV FWLS <15%) linked to poor LV strain response; baseline TR grade ≈1.9 (0–3) \| |
| --- | --- | --- | --- | --- | --- | --- | --- | --- | --- | --- | --- | --- | --- | --- | --- | --- | --- | --- | --- | --- | --- | --- | --- | --- | --- | --- | --- | --- | --- | --- | --- | --- | --- | --- | --- | --- | --- | --- | --- | --- | --- | --- | --- | --- | --- | --- | --- | --- | --- | --- | --- | --- | --- | --- | --- | --- | --- | --- | --- | --- | --- | --- | --- | --- | --- | --- | --- | --- | --- | --- | --- | --- | --- | --- | --- | --- | --- | --- | --- | --- | --- | --- | --- | --- | --- | --- | --- | --- | --- | --- | --- | --- | --- | --- | --- | --- | --- | --- | --- | --- | --- | --- | --- | --- | --- | --- | --- | --- | --- | --- | --- | --- | --- | --- | --- | --- | --- | --- | --- | --- | --- | --- | --- | --- | --- | --- | --- | --- | --- | --- | --- | --- | --- | --- | --- | --- | --- | --- | --- | --- | --- | --- | --- | --- | --- | --- | --- | --- | --- | --- | --- | --- | --- | --- | --- | --- | --- | --- | --- | --- | --- | --- | --- | --- | --- | --- | --- | --- | --- | --- | --- | --- | --- | --- | --- | --- | --- | --- | --- | --- | --- | --- | --- | --- | --- | --- | --- | --- | --- | --- | --- | --- | --- | --- | --- | --- | --- | --- | --- | --- | --- | --- | --- | --- | --- | --- | --- | --- | --- | --- | --- | --- | --- | --- | --- | --- | --- | --- | --- | --- | --- | --- | --- | --- | --- | --- | --- | --- | --- | --- | --- | --- | --- | --- | --- | --- | --- | --- | --- | --- | --- | --- | --- | --- | --- | --- | --- | --- | --- | --- | --- | --- | --- | --- | --- | --- | --- | --- | --- | --- | --- | --- | --- | --- | --- | --- | --- | --- | --- | --- | --- | --- | --- | --- | --- | --- | --- | --- | --- | --- | --- | --- | --- | --- | --- | --- | --- | --- | --- | --- | --- | --- | --- | --- | --- | --- | --- | --- | --- | --- | --- | --- | --- | --- | --- | --- | --- | --- | --- | --- | --- | --- | --- | --- | --- | --- | --- | --- | --- | --- | --- | --- | --- | --- | --- | --- | --- | --- | --- | --- | --- | --- | --- | --- | --- | --- | --- | --- | --- | --- | --- | --- | --- | --- | --- | --- | --- | --- | --- | --- | --- | --- | --- | --- | --- | --- | --- | --- | --- | --- | --- | --- | --- | --- | --- | --- | --- | --- | --- | --- | --- | --- | --- | --- | --- | --- | --- | --- | --- | --- | --- | --- | --- | --- | --- | --- | --- | --- | --- | --- |

S2.5 Statistical analysis

We provide the following additional details on data synthesis. The studies reported the effect of surgical therapy in different ways: either in the form of baseline and after-treatment means at the group level or the baseline means and the change from baseline to a group level. If the change from baseline was not reported, it was calculated as a difference between before (baseline) and after treatment means. The within-group correlation between before-treatment and after-treatment was assumed to equal across groups and studies and was estimated based on the extracted SD of the change when the SD for the baseline and after- treatment value was also provided. When the SD was not given, but standard error (SE) or confidence interval was available, we calculated the SD based on it (e.g. multiplying by the square root of the sample size for SE or using the t-distribution value for confidence interval based on the given confidence level). To calculate study MDs, the sample size, the mean, and the corresponding standard deviation (SD) were extracted from each study (in each group separately). The mean values of the control group were subtracted from the mean values of the experimental group. If the quartiles were given instead of the mean, SD, or SEM, for estimating the mean and standard deviation from the quartiles, Lou and Shi methods were used, as implemented in the used meta R package. To calculate study proportions and pooled proportions, the number of all patients and those with the event of interest was extracted from each study.
