# Supplementary figures and images for: Assessing the impact of transcatheter edge-to-edge repair on reverse remodeling in secondary mitral regurgitation: a systematic review and meta-analysis
Source: Front Cardiovasc Med. 2026 Jan 30;12:1714337. doi: 10.3389/fcvm.2025.1714337 (PMC12901449; doi:10.3389/fcvm.2025.1714337)

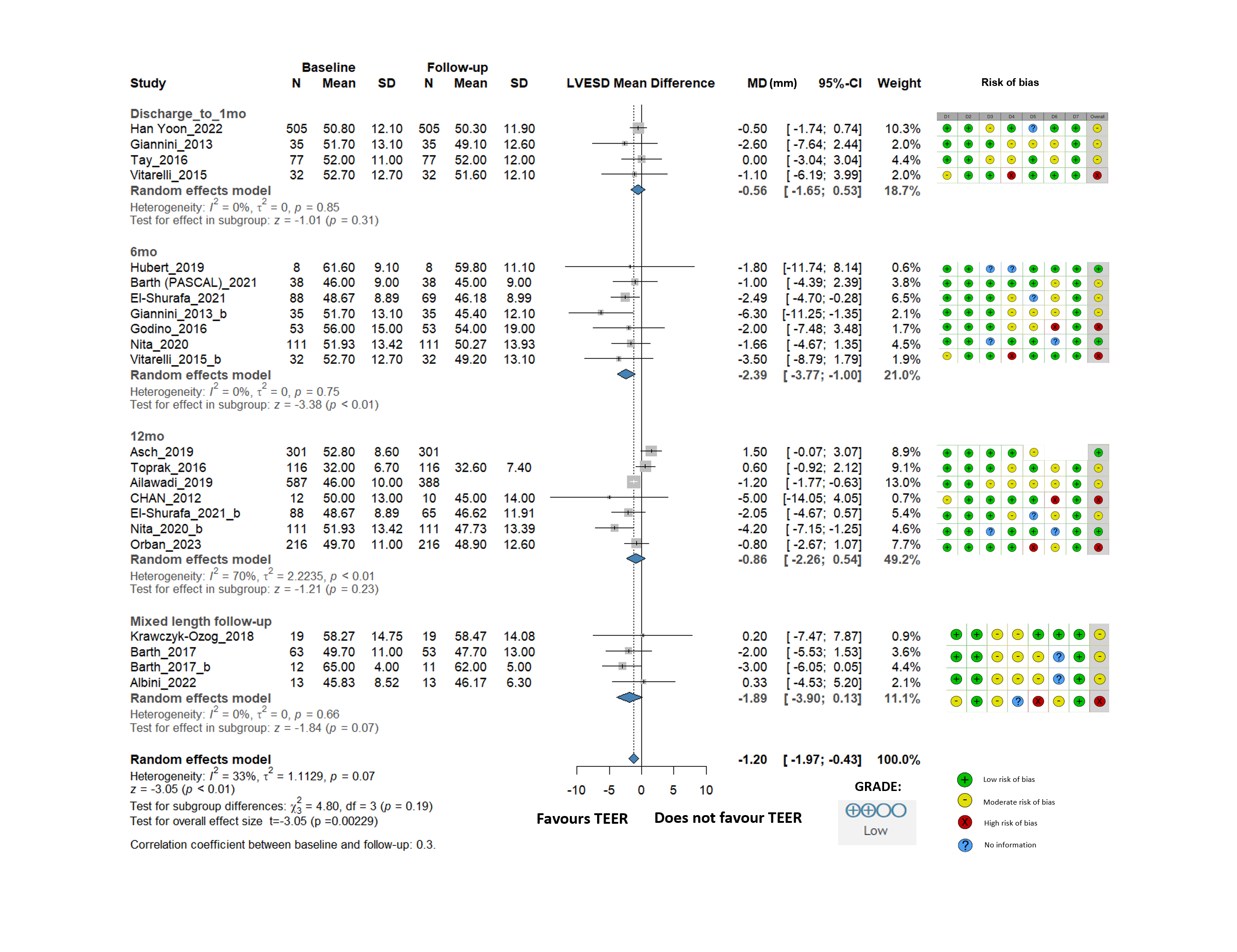

Supplement: Supplementary file 2 [file Image1.tif]

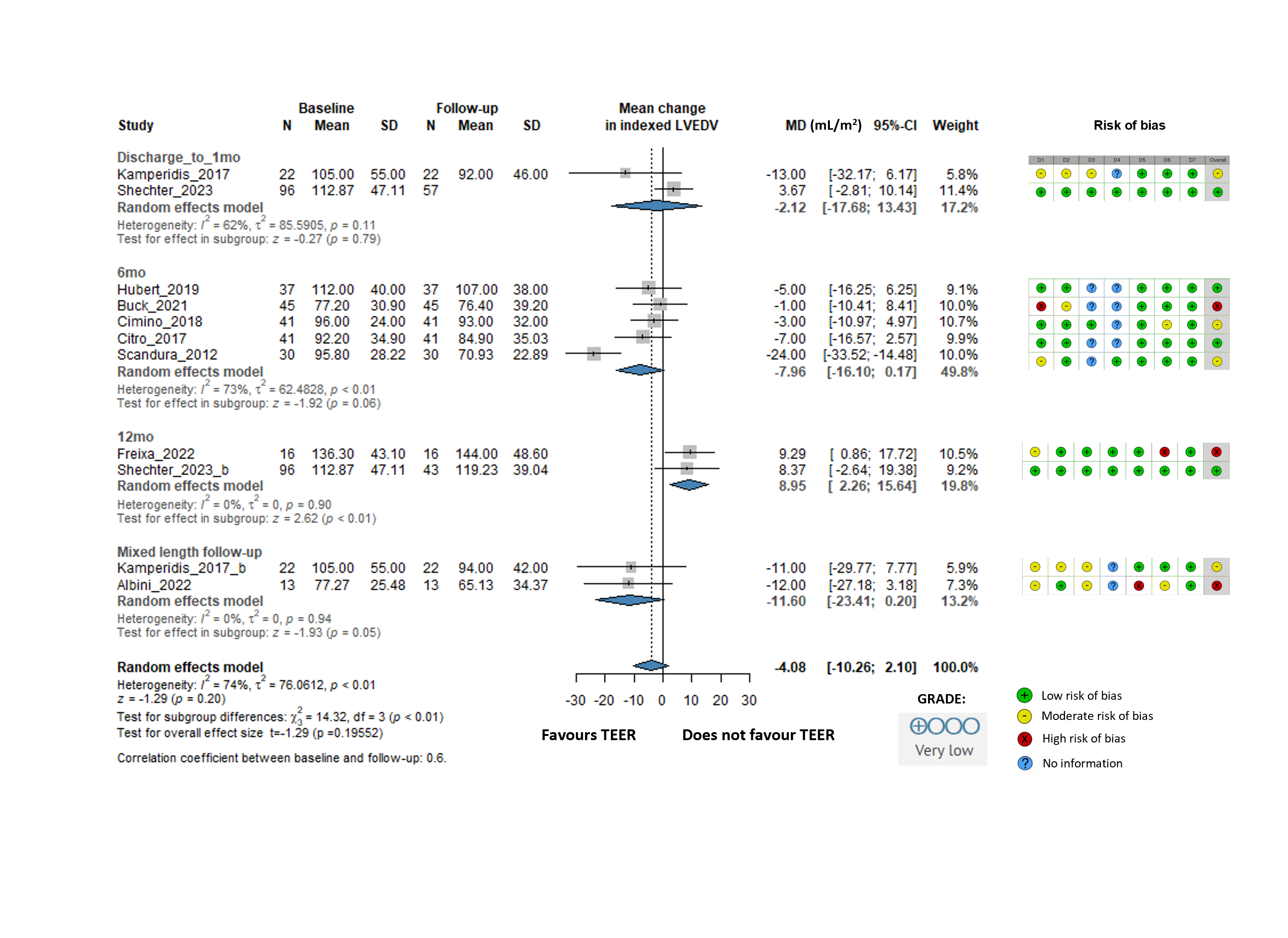

Supplement: Supplementary file 3 [file Image2.tif]

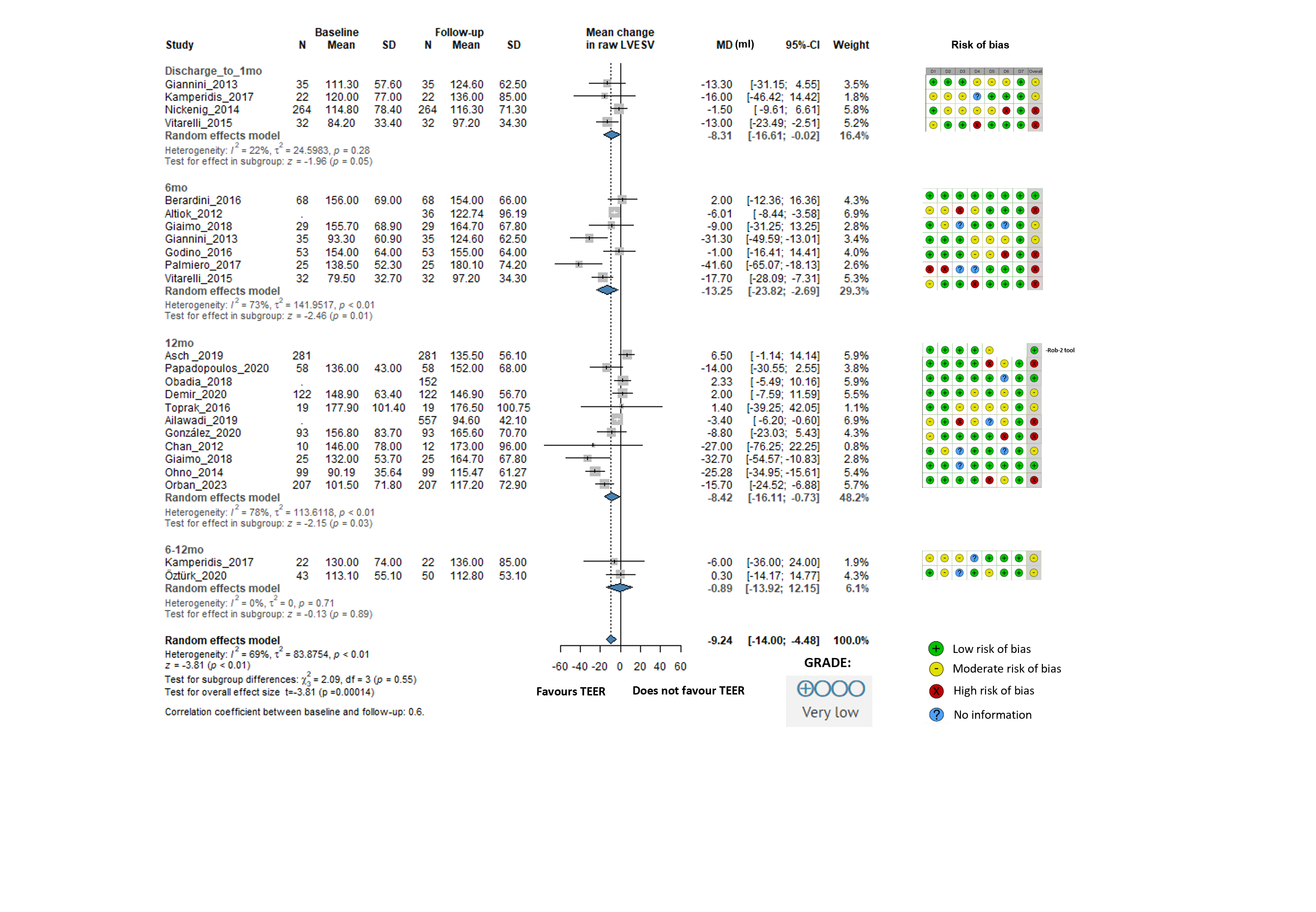

Supplement: Supplementary file 4 [file Image3.tif]

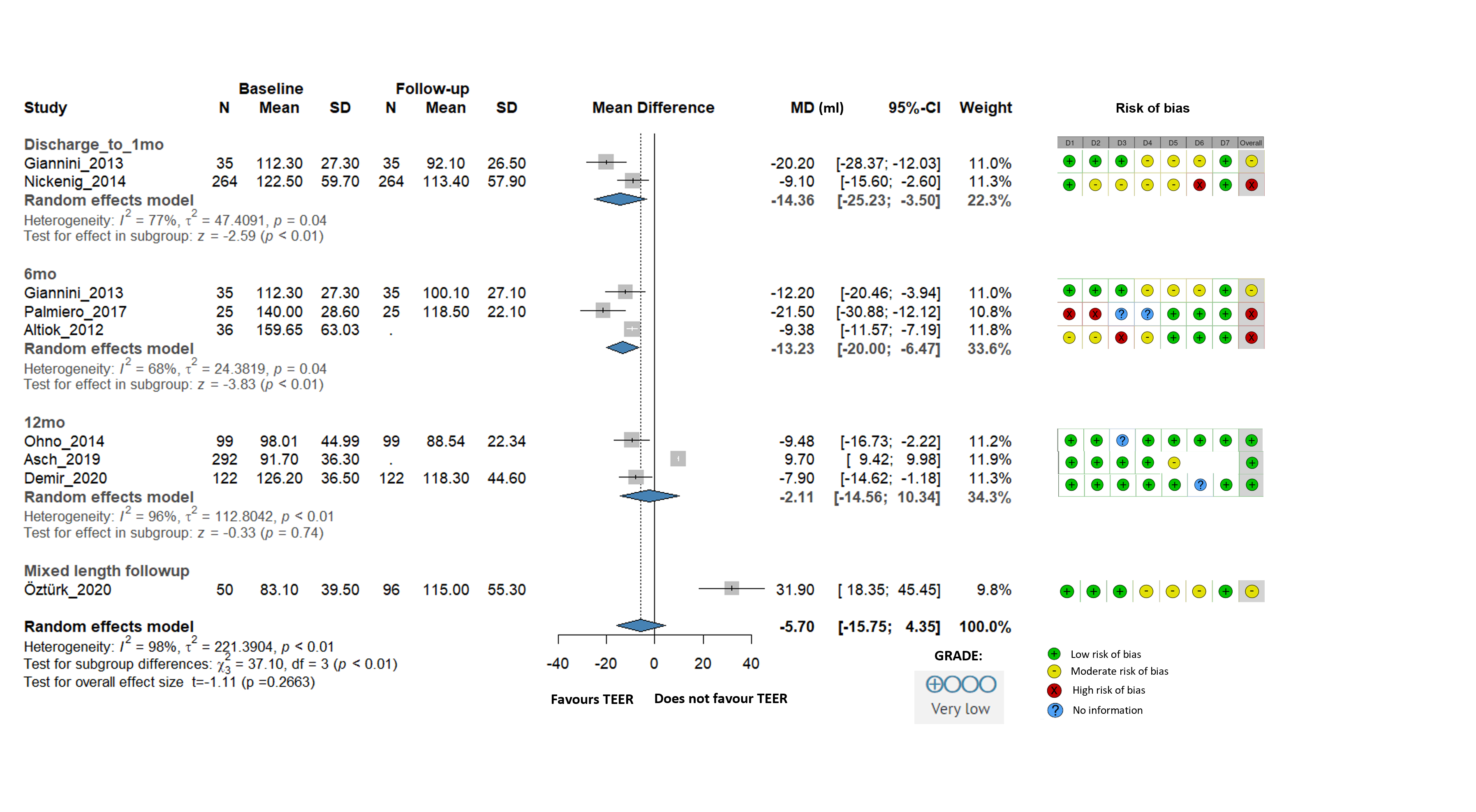

Supplement: Supplementary file 5 [file Image4.tif]

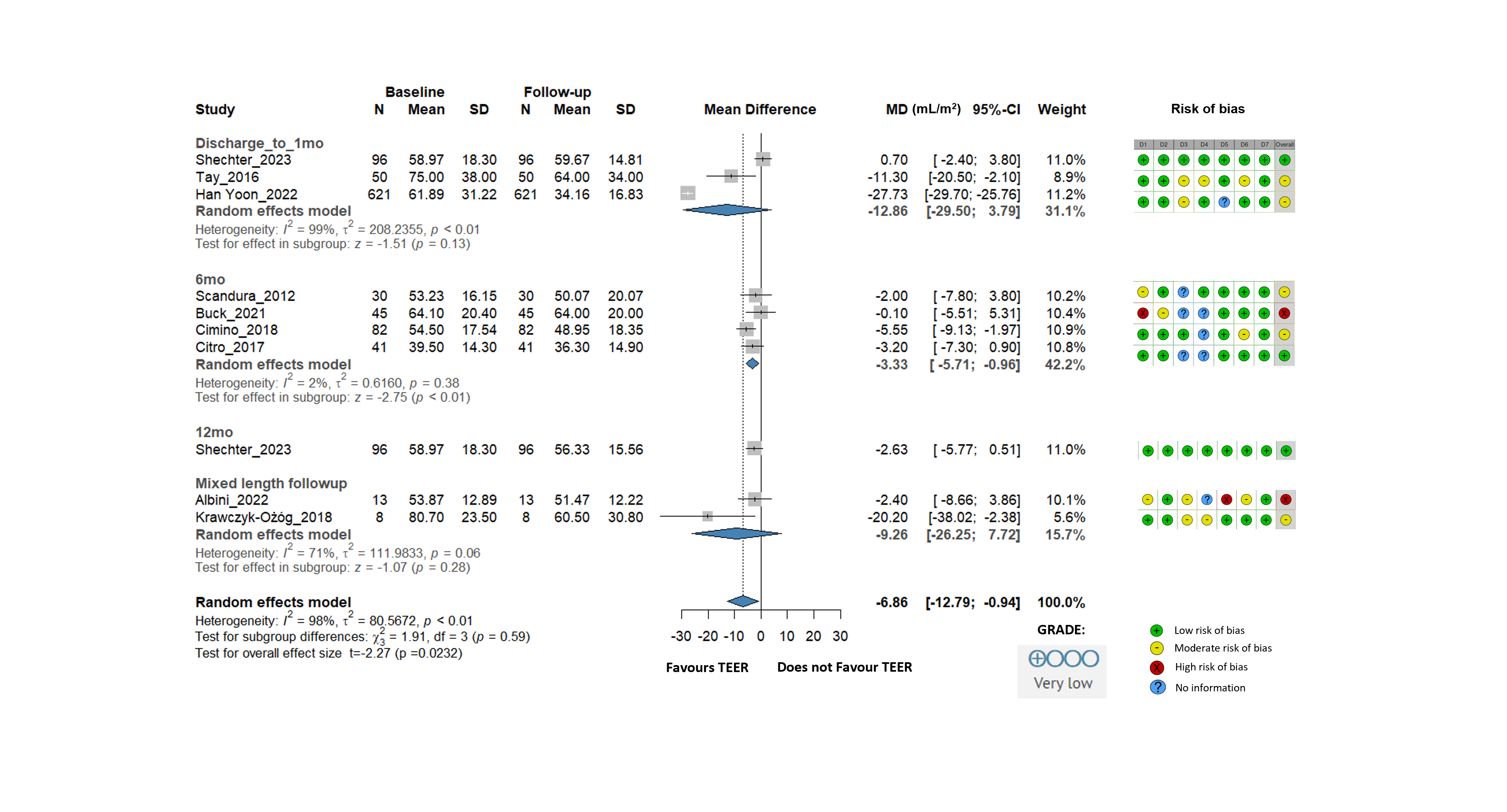

Supplement: Supplementary file 6 [file Image5.tif]

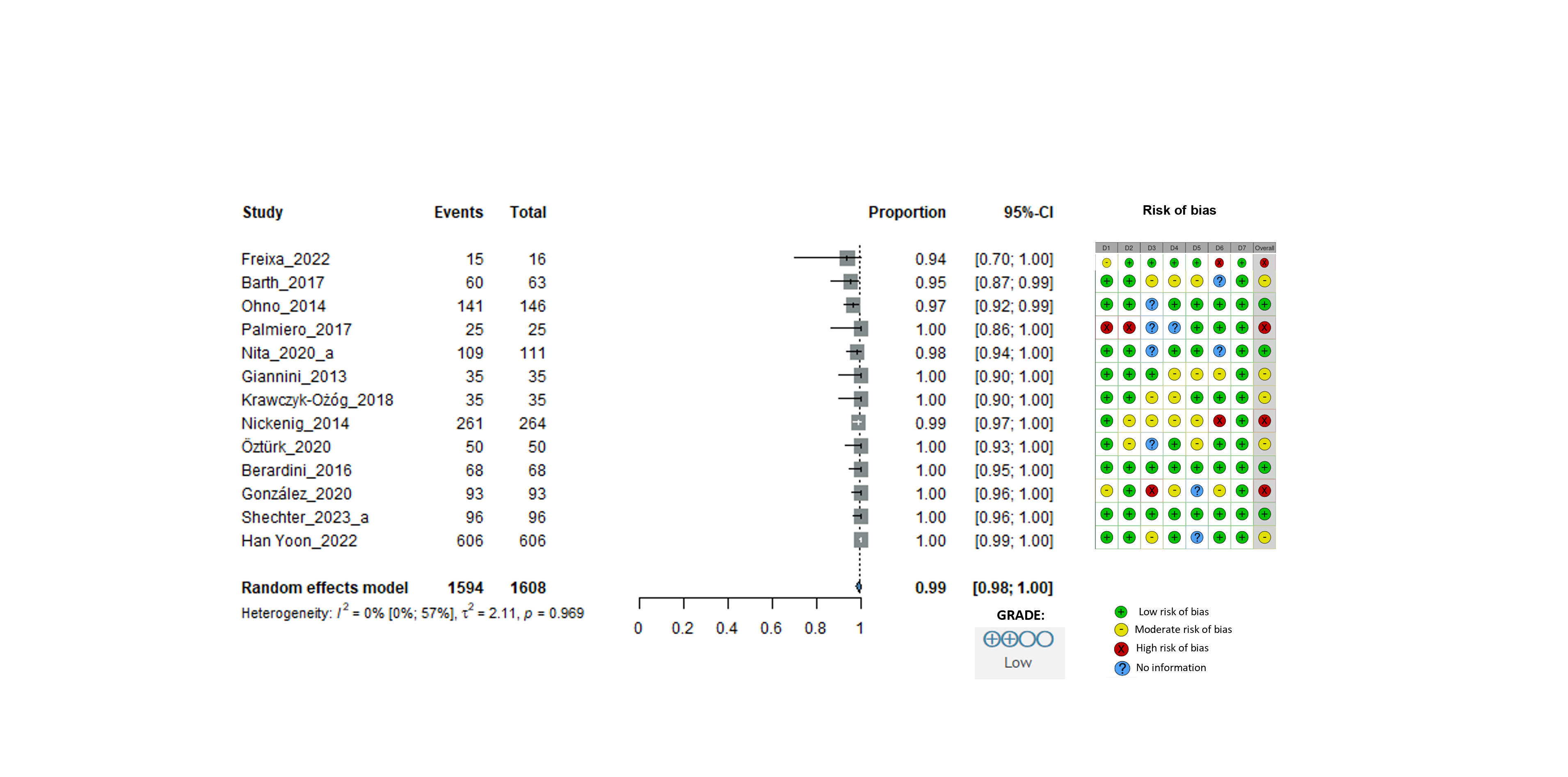

Supplement: Supplementary file 7 [file Image6.tif]

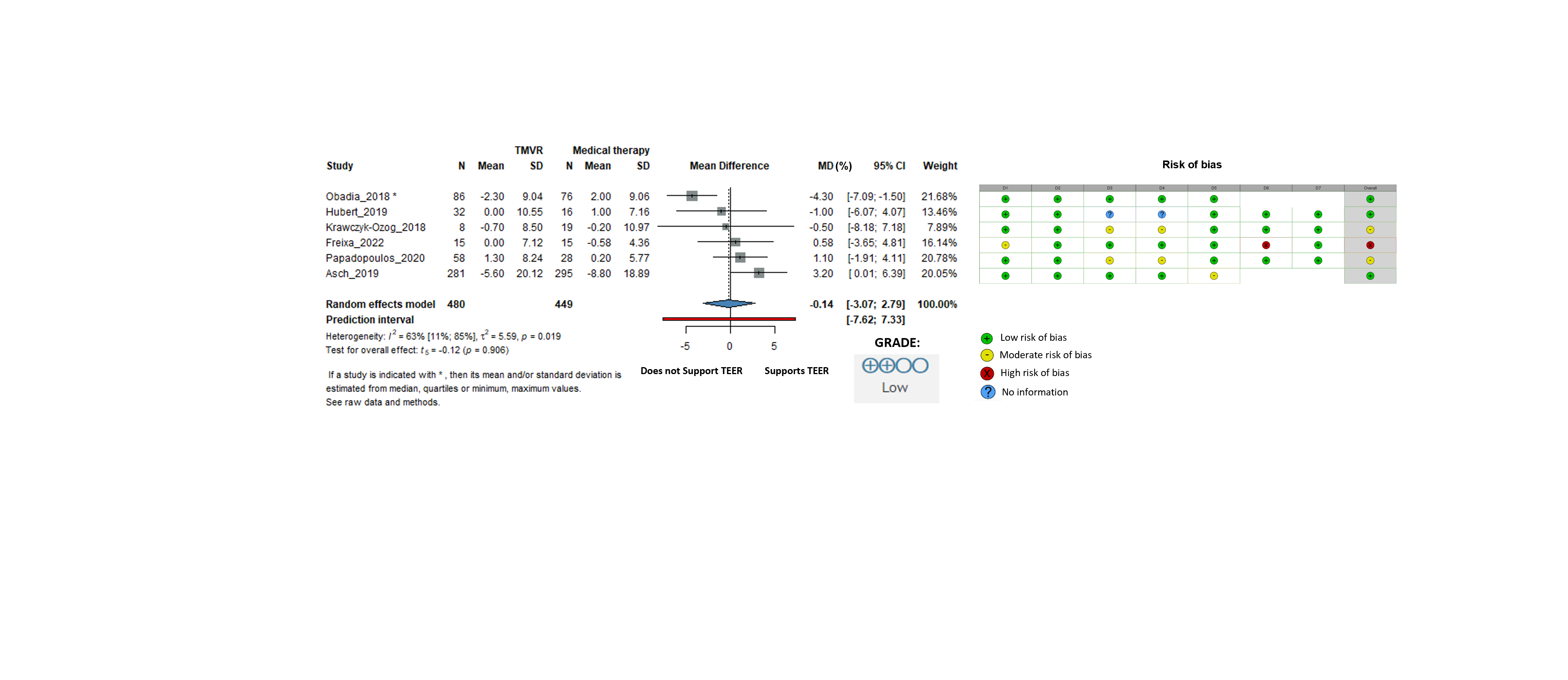

Supplement: Supplementary file 8 [file Image7.tif]

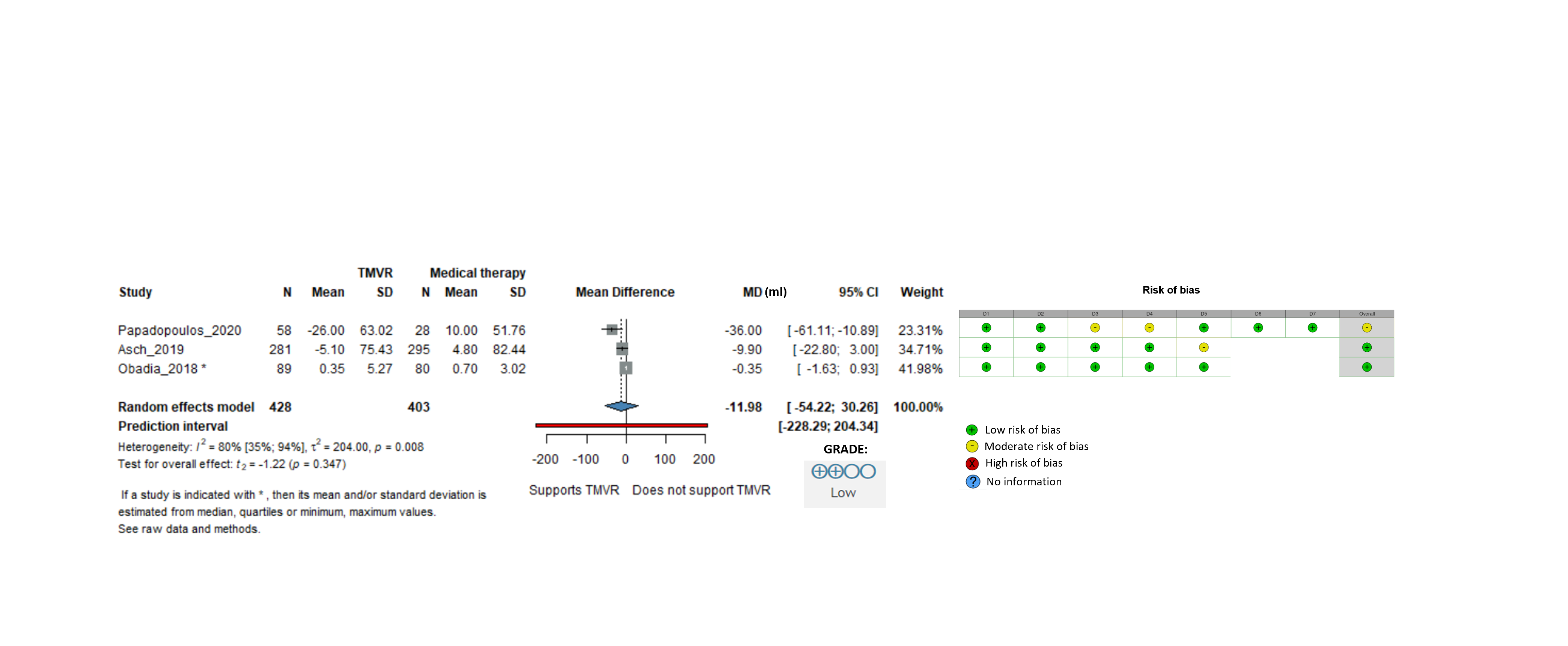

Supplement: Supplementary file 9 [file Image8.tif]

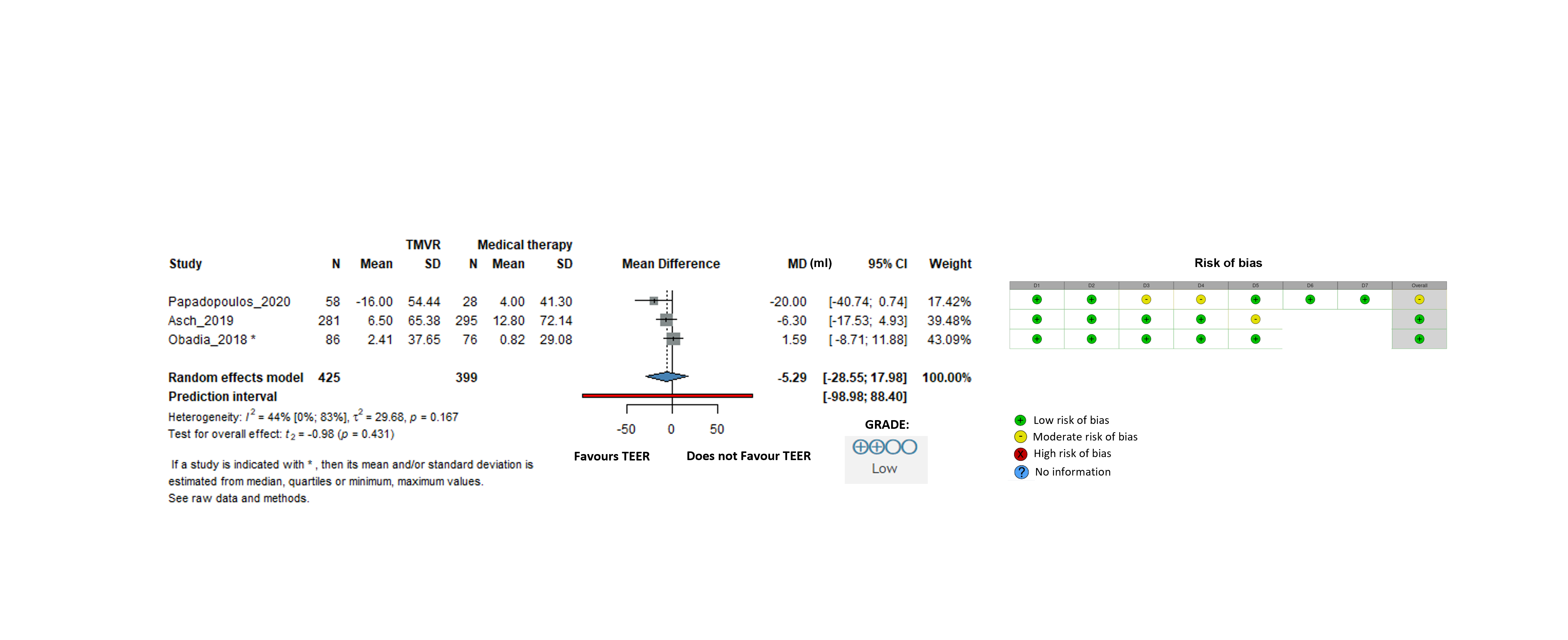

Supplement: Supplementary file 10 [file Image9.tif]
